# Hapten Design and Monoclonal Antibody to Fluoroacetamide, a Small and Highly Toxic Chemical

**DOI:** 10.3390/biom10070986

**Published:** 2020-07-01

**Authors:** Ling Yang, Xiya Zhang, Dongshuai Shen, Xuezhi Yu, Yuan Li, Kai Wen, Jianzhong Shen, Zhanhui Wang

**Affiliations:** 1 Beijing Advanced Innovation Center for Food Nutrition and Human Health, College of Veterinary Medicine, China Agricultural University, Beijing Key Laboratory of Detection Technology for Animal-Derived Food Safety, and Beijing Laboratory for Food Quality and Safety, Beijing 100193, China; yl16@cau.edu.cn (L.Y.); shends1992@cau.edu.cn (D.S.); yuxuezhi@cau.edu.cn (X.Y.); b20183050469@cau.edu.cn (Y.L.); wenkai@cau.edu.cn (K.W.); sjz@cau.edu.cn (J.S.); 2 College of Food Science and Technology, Henan Agricultural University, Zhengzhou 450002, China; zhangxiya@henau.edu.cn

**Keywords:** fluoroacetamide, hapten design and synthesis, antibodies, toxins, immunoassay

## Abstract

Fluoroacetamide (FAM) is a small (77 Da) and highly toxic chemical, formerly used as a rodenticide and potentially as a poison by terrorists. Poisoning with FAM has occurred in humans, but few reliably rapid detection methods and antidotes have been reported. Therefore, producing a specific antibody to FAM is not only critical for the development of a fast diagnostic but also a potential treatment. However, achieving this goal is a great challenge, mainly due to the very low molecular weight of FAM. Here, we design two groups of FAM haptens for the first time, maximally exposing the fluorine or amino groups, with the aid of linear aliphatic or phenyl-contained spacer arms. Interestingly, whereas the hapten with fluorine at the far end of the hapten did not induce an antibody response to FAM, the hapten with an amino group at the far end and phenyl-contained spacer arm triggered a significantly specific antibody response. Finally, a monoclonal antibody (mAb) named 5D11 was successfully obtained with an IC_50_ value of 97 μg mL^−1^ and negligible cross-reactivities to the other nine functional and structural analogs.

## 1. Introduction

Fluoroacetamide (FAM) is a fluorine compound with tremendous toxicity, formerly used as a rodenticide and insecticide, which inhibits the citrate metabolism by blocking its transport into mitochondria and its breakdown by aconitase in vivo [1,2]. The mean lethal dose of FAM varies from 0.05 mg/kg for dogs to 150 mg/kg for possums, while 50 mg/kg is considered to be a lethal dose for humans [3,4,5]. The serum FAM in 65 cases of acute toxicosis patients had been monitored continuously; the peak value of the serum FAM appeared on the third hour and varied from 0.81 to 3.65 mg/L [6,7]. Nowadays, almost all countries have banned the use of FAM because of its high and acute toxicity. However, it is still inappropriately or illegally used for its low cost and high effectiveness, especially in developing countries. It has been reported that the occurrence of FAM poisoning cases is on the rise, including suicide, poisoning, or consumption of contaminated food [8]. More importantly, FAM is considered a potential terror agent to national security because it acts rapidly and can cause death within hours [2,9]. Therefore, guidelines and tools for a timely identification, determination, and subsequent treatment of FAM poisoning are urgently needed. However, only a few reported rapid diagnostic methods for its detection have been published, and no reliably proven antidotes are available for FAM poisoning in both the academic and commercial fields [10].

**Figure 1:**
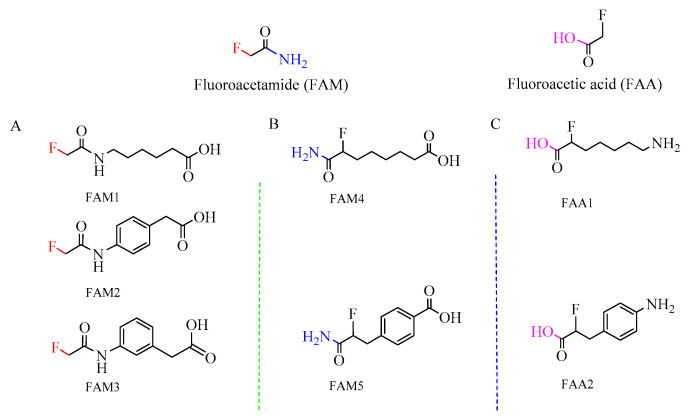
The chemical structures of the fluoroacetamide (FAM) and fluoroacetic acid (FAA) haptens. (**A**) The group of FAM haptens maximally exposing fluorine; (**B**) the group of FAM haptens maximally exposing the amino group; (**C**) the group of haptens derived from FAA.

Instrument-based methods have been developed and applied to the detection of FAM, including gas chromatography (GC), gas chromatography-tandem mass spectrometry (GS–MS), electrochemical detection, and surface plasmon resonance (SPR)-based assay [8,9,11,12,13,14,15]. Although the instrumental methods are precise, sensitive, and accurate, they usually require expensive equipment, a complicated sample processing, and trained operators, which limits their distribution and does not provide us with suitable devices for the on-site monitoring of poisoning. Immunoassays based on antigen–antibody specific interactions are promising for the clinical diagnose of poisons because these methods are rapid, easy to use, and low-cost. The antibody is the critical biomolecule because it governs the sensitivity and specificity of an immunoassay. In addition, the antibody can also be used as an effective antidote for chemical poisoning such as in the case of digoxin and drugs of abuse [16,17]. The production of a sensitive and specific antibody to FAM is challenging, mainly due to its very small molecular weight of 77 Da, which may not be enough to make even a single epitope (Figure 1). Under such circumstances, a derivatization step before analysis is usually employed for indirect detection, for example, nitrofuran metabolites, acrylamide, and histamine [18,19,20]. Without derivatization, the 50% inhibition concentration (IC_50_) of ELISA for these linear and straightforward molecules is usually in the range of specific binding of μg mL^−1^. Detailed information about the antibodies for typical very small molecules is summarized in Table S1. FAM lacks an active group for efficiently reacting with derivatization reagents like nitrobenzaldehyde in the case of nitrofuran metabolites [20]. Thus, producing specific antibodies to FAM is an inevitable strategy to develop an immunoassay for the direct detection of FAM. As a small molecule, FAM is not capable of eliciting an immune response by itself, unless FAM (or its structural analogs named haptens) is chemically linked to a carrier protein to form a complete antigen.

In this study, two groups of FAM haptens were synthesized, maximally exposing fluorine or the amino group, with the aid of linear aliphatic or phenyl-contained spacer arms, as shown in Figure 1A,B. We evaluated and discussed the effectiveness of these haptens in the induction of antibody production to FAM, mainly focusing on the structure of haptens. We also evaluated the function of the benzoylformic acid in the spacer arm, focusing on whether it guaranteed specific antibody formation, by designing fluoroacetic acid (FAA) haptens (Figure 1C). Finally, one highly specific and high-affinity monoclonal antibody (mAb) to FAM was generated, which could be a potential reagent for assays and treatment. This work also provided useful clues to produce high-quality antibodies against small molecules.

## 2. Materials and Methods

### 2.1. Hapten Synthesis and Conjugate Preparation

**Scheme 1:**
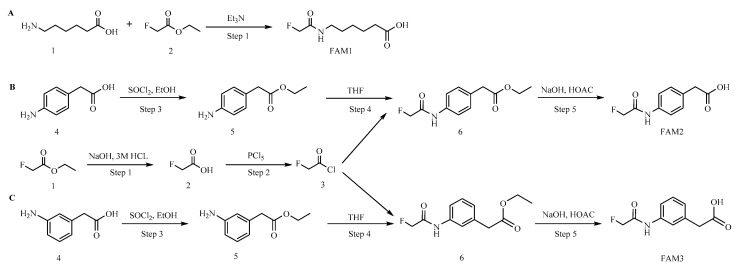
Synthetic routes of FAM1 (**A**), FAM2 (**B**), and FAM3 (**C**).

**Scheme 2:**
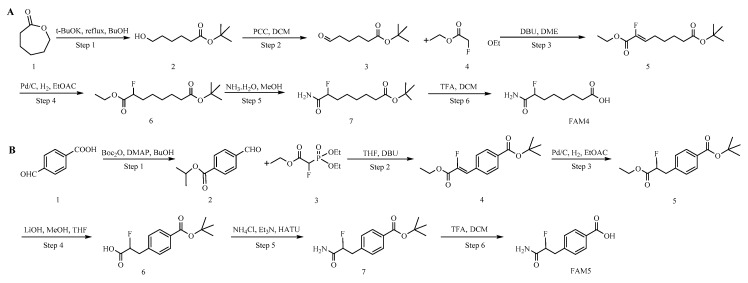
Synthetic routes of FAM4 (**A**) and FAM5 (**B**).

**Scheme 3:**
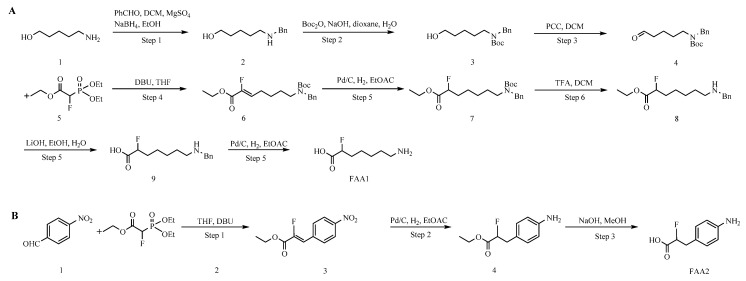
Synthetic routes of FAA1 (**A**) and FAA2 (**B**).

Two groups of FAM haptens and one group of FAA haptens (Figure 1) were designed and synthesized. The synthetic routes are shown in Scheme 1, Scheme 2, and Scheme 3. The synthetic procedure and characterization of haptens except FAM5 are given in the Supporting Information.

FAM was purchased from Dr. Ehrenstorfer Gmbh (Ausburg, Germany) and all other materials were purchased from commercial sources and used without further purification. The synthetic route of hapten FAM5 was presented in Scheme 2 in 6 steps. Briefly, a solution of compound 1 (22.52 g, 150 mmol) and DMAP (9.20 g, 75 mmol) was added in *t*-BuOH (300 mL) and cooled to 0 °C. Boc_2_O (65.47 g, 300 mmol) was added, and the mixture was heated at 30 °C for 8 h, then cooled to room temperature (RT) and poured onto water (1000 mL). The mixture was extracted, concentrated, dried, purified, and obtained a yellow sold as compound 2 (21.20 g, 68%). Compound 2 (18.20 g, 88.2 mmol), compound 3 (32.00 g, 132 mmol), and DBU (20.20 g, 132 mmol) were resolved in THF (120 mL) and stirred at RT for 1 h. To the reaction mixture was added 1 N HCl (200 mL), and then the solution was extracted, dried, and purified and obtained compound 4. Palladium on carbon (10%, 3.00 g) was added to a solution of compound 4 (20.30 g, 69 mmol) in EtOAc (300 mL). The reaction mixture was hydrogenated under hydrogen bloom for 16 h. The reaction mixture was filtered to remove the palladium on the carbon. The filtrate was concentrated and purified, and compound 5 was thus obtained. Compound 5 (14.20 g, 48 mmol) in MeOH (60 mL) and THF (20 mL) were added to a LiOH aqueous solution (2.42 g, 57.6 mmol in 20 mL of water) and stirred at RT for 16 h, and then concentrated and purified to obtain compound 6. Compound 6 (4.10 g, 15.3 mmol), triethylamine (4.64 g, 46 mmol), ammonium chloride (2.50 g, 46.7 mmol), and HATU (7.60 g, 20 mmol) were added to DCM (50 mL). The reaction mixture was stirred at RT for 16 h; then the separated organic layer was extracted, concentrated, and purified to obtain compound 7. Compound **7** (3.70 g, 13.8 mmol) in DCM (30 mL) was added to TFA (10 mL) and stirred at RT for 16 h. The mixture was concentrated, filtered, and FAM5 was collected as a white solid (2.80 g, 96%). HRMS (*m/z*) calc. for C_10_H_10_FNO_3_^(−)^ 211.06, found 210.05, ^1^H NMR (400 MHz, DMSO-*d_6_*) δ 2.51-3.30 (m, 2H), 5.04-5.19 (m, 1H), 7.38 (d, *J* = 8.0 Hz, 2H), 7.46 (s, br, 1H), 7.59 (s, br, 1H), 7.88 (d, *J* = 8.0 Hz, 2H), 12.87 (s, 1H).

Keyhole limpet hemocyanin (KLH) and bovine serum albumin (BSA) acquired from Sigma-Aldrich (St. Louis, MO, USA) were used as carriers for the immunogens and coating antigens, respectively. The preparation of the hapten–protein conjugates is described in the Supporting Information.

### 2.2. ELISA Method Development

The indirect non-competitive ELISA (nc-ELISA) was used to determine the titers of antisera, hybridomas supernatant, and resultant mAb. The indirect competitive ELISA (ic-ELISA) was used to determine the affinity and specificity of mAbs. A four-parameter logistic equation was used to fit the ic- ELISA data.

Y = (A − D)/[1 + (X/C)^B^] + D, (1)

where *A* is the response at high asymptote, *B* is the slope factor, *C* is the concentration corresponding to 50% specific binding (IC_50_), *D* is the response at low asymptote, and *X* is the calibration concentration. The detailed ELISA methods were performed as described in the Supporting Information.

### 2.3. Mice Immunization and Antisera Analysis

The BALB/c mice were immunized, and the antisera titer and affinity were analyzed by both nc-ELISA and ic-ELISA according to our reported procedure [21] and described in the Supporting Information.

### 2.4. mAb Production

Splenocytes from immunized mice were fused with SP2/0 myeloma cells using PEG purchased from Sigma-Aldrich (St. Louis, MO, USA) as a fusing reagent. The fusion, cell cultivation, and cloning procedures are described in the Supporting Information.

### 2.5. Cross-Reactivity Determination

The specificity of mAb in the ic-ELISA was performed using nine structural/functional FAM analogs, including bromoacetic acid, chloroacetamide, chloroacetic acid, difluoroacetic acid, FAA, thiosemicarbazide, 1-chloro-3-fluoroisopropanol, 1,3-difluoro-2-propanol, and 2,2,2-trifluoroacetamide. The tested compounds (10–10000 μg/mL) were deployed to the icELISA procedure, as described above for FAM. The cross-reactivities of mAb (CR) values were then calculated as:

CR (%) = (IC_50-FAM_/IC_50-analogues_) × 100%. (2)

## 3. Results

### 3.1. Hapten Design and Conjugate Preparation

Both linear aliphatic-contained and phenyl-contained spacer arms were used in the hapten design of FAM. Since the particular structure of FAM is fluorine, the spacer arm of hapten should be preferentially linked to the amino group, exposing the fluorine at best to generate the specific antibody. As shown in Figure 1A, efforts were made to design the hapten, exposing fluorine with different spacer arms as much as possible, which was expected to enhance the immune response. For comparison, the spacer arms are also designed to link the carbon adjacent to fluorine, exposing the amino group, again with the consideration of the small FAM (Figure 1B). Thus, two groups of FAM haptens are designed, and the synthesis routes are shown in Scheme 1 and Scheme 2. The detailed synthetic routes and characterization of FAM haptens are provided in the Supporting Information (Figure S1A–E).

**Figure 2:**
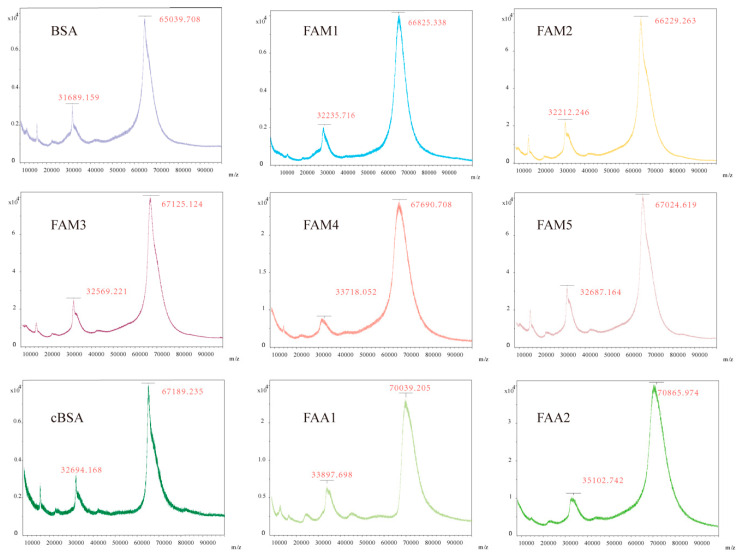
MALDI-TOF-MS results of bovine serum albumin (BSA), cBSA, FAM1-BSA~FAM5-BSA, FAA1-BSA, and FAA2-BSA. The calculated hapten/protein ratios were 9.3, 5.6, 9.8, 13.9, 9.4, 17.5, and 20.0, respectively. FAM1~FAM5 were calculated with BSA, whereas FAA1 and FAA2 were calculated with cBSA.

To conjugate the haptens to the carrier protein, the carboxylic acids of haptens were activated with *N*-hydroxysulfosuccinimide and dicyclohexylcarbodiimide, and then treated with KLH as immunogens and BSA as coating antigens. The BSA conjugates are characterized by matrix-assisted laser desorption/ionization-time of flight mass spectrometry (MALDI-TOF-MS) (Bruker, Rheinstetten, Germany), as shown in Figure 2. A noticeable shift in the peak maximum of the protein conjugate in comparison with the control protein is observed, demonstrating that the haptens had been conjugated to a carrier protein. Since the hapten–carrier ratio could influence the antibody response in animals and the subsequent antibody test, the ratios of hapten–BSA were then calculated to be 5.6 to 13.9, which are optimal to be used as immunogens [22].

### 3.2. Mice Immunization and Antisera Analysis

Each hapten–KLH conjugate was used to immunize a set of eight BALB/c mice a total of three times at 28-day intervals, minimizing the risk of obtaining results overly biased by any individual mouse. The effectiveness of haptens to antibody response was evaluated by an antibody titer and inhibition ratio determined on the homologous and heterologous coating antigens (10 mg/mL, 1:10,000 dilution) by the nc-ELISA and ic-ELISA, respectively (Supporting Information). In the study, an antibody titer was defined as the dilution of antisera with an OD_max_ between 1.5 and 2.0 measured using Multiskan FC microplate reader (Thermo Scientific). In contrast, antibody affinity was expressed as the inhibition ratio calculated according to an equation (Supporting Information). In short, a higher antibody dilution or lower inhibition ratio indicates a higher antibody response.

**Figure 3:**
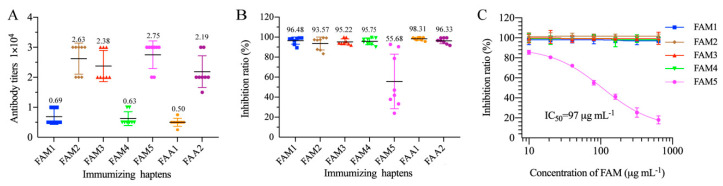
Evaluation of antibody titer and affinity. (**A**) Antibody titers of murine antisera tested by their homologous ELISA at the last immunization. The titer is defined to be the dilution of antisera with an ODmax between 1.5 and 2.0 with the coating antigen concentration of 1 μg/mL. Values are means ± SDs, *n* = 8. (**B**) Inhibition ratios representing antibody affinities were measured by the best-paired coating antigens for FAM at the last immunization. The inhibition ratio is calculated according to an equation described in the Supporting Information. Values are means ± SDs, *n* = 8. (**C**) The competitive standard curves of the mAb5D11 for FAM pairing with the heterologous coating antigen FAM1-BSA (1 μg/mL). Values are means ± SDs, *n* = 3.

As shown in Figure 3A and Table S2, a significant difference in the antibody titer from these haptens was observed. The haptens containing linear aliphatic spacer arms, FAM1 and FAM4, showed the lowest recognition to coating antigens with the titers of about 6000, implying that the linear aliphatic spacer arm of hapten is not enough to induce a strong antibody response, possibly because of its simple structure and consequently low immunogenicity. The other three phenyl-contained haptens, FAM2, FAM3, and FAM5, induced a more robust antibody response with similar titers, all above 30,000, demonstrating that a bulky spacer arm such as phenyl is beneficial to assist the small molecule in inducing a strong antibody response.

The performance of antisera to the small molecule should be evaluated not only by the antibody titer but also antibody affinity. The latter is practically more critical in the fields of bioanalysis and biomedicine. It can be observed in Figure 3B and Table S2 that only antisera derived from hapten FAM5 showed a significant recognition ability to free FAM. Six antisera of eight mice immunized by FAM5-KLH showed obvious inhibition ratios of 23–78% at the concentration of 500 μg/mL FAM. The antisera from other haptens all exhibited above 90% inhibition ratios, meaning that quite a small amount of specific antibody to FAM in the mice was induced. The results indicate that the titers of antisera from other haptens to coating antigens are possibly attributed to the high binding ability of antisera to whole hapten–protein conjugates. Still, only FAM, thus resulting in difficult displacement by free FAM.

The particular structure of FAM5 contributes to the formation of a specific antibody to FAM. In Figure 1A,B, another difference can be observed between FAM2, FAM3, and FAM5: the methylene position of the spacer arm beside the different position of fluorine. In detail, the *p*- or *m*- phenylacetic acids are linked to the amino group of FAM, generating FAM2 and FAM3, respectively. In contrast, benzoylformic acid is linked to the carbon that fluorine connected, producing FAM5. To evaluate whether the introduction of the benzoylformic acid could increase the antibody titer and affinity against the small molecule, we then designed another two haptens that started from the FAA, obtaining the FAA1 and FAA2 haptens shown in Figure 1C (the synthetic procedure is described in Scheme 3). To prevent the possible linkage of the usually employed carboxyl group of hapten to a carrier protein, the right end of the haptens was designed with the amino groups (Figure 1C). After characterization, conjugation with the cationic carrier protein, and immunization (Figure S1F–G and Figure 2), the results showed that the two new haptens triggered similar antibody responses with the linear aliphatic-contained FAM1 and FAM4, and phenyl-contained FAM2 and FAM3 (Figure 3 and Table S2). The linear aliphatic containing FAA1 or phenyl-contained FAA2 did not induce any specific antibody to free FAA, although significant antibody titers to their homologous coating antigens were observed. The results imply that the use of benzoylformic acid as a spacer arm does not always guarantee the induction of specific antibodies to a small molecule. In addition, the higher antibody titers induced by the phenyl-contained hapten FAA2 compared with those induced by the linear aliphatic-contained hapten FAA2 suggest that the phenyl-contained hapten design strategy could at least guarantee the production of a high antibody titer.

### 3.3. mAb Production and Characterization

**Figure 4:**
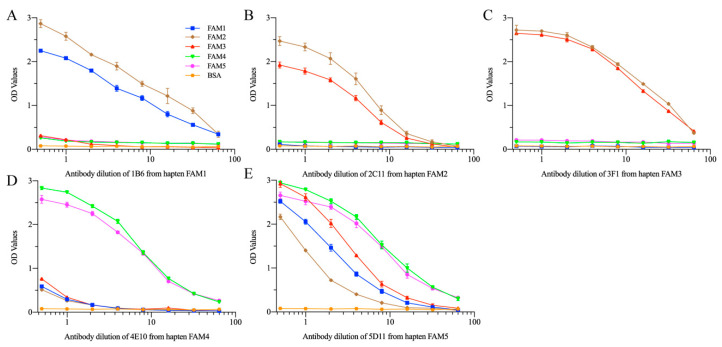
Monoclonal antibodies (mAbs) of 1B6 (**A**), 2C11 (**B**), 3F1 (**C**), 4E10 (**D**), and 5D11 (**E**) dilution curves combined with homologous and heterologous FAM1-BSA~FAM5-BSA coating antigens (1 μg/mL). For clarity, the first number of each mAb name is consistent with the order of haptens shown in Figure 1.

Although only the antisera from FAM5 showed specific recognition of free FAM, mice with the highest antisera titers from each hapten were used in the hybridoma preparation since, in theory, specific mAbs could still be obtained by an exhausting screening step. Moreover, unlike the antisera composed by a mixed antibody that can bind many epitopes, a single mAb can, in principle, only bind to one epitope, which could insert into the recognition profiles of one certain mAb [23]. Finally, five mAbs, named 1B6, 2C11, 3F1, 4E10, and 5D11, were obtained, and their isotypes were tested and are shown in Table S3. For clarity, the first number of each mAb name is consistent with the order of haptens shown in Figure 1. Subsequently, five mAbs and five coating antigens were paired into 25 combinations to evaluate and optimize the performances of those mAbs in ELISA when the coating protein BSA was used as the control. The recognition profiles of 25 mAbs–antigens combinations are shown in Figure 4. Every mAb could bind its homologous coating antigen. It could not bind BSA, meaning that each hapten has successfully induced the generation of the antibody that could specifically recognize the epitopes partly formed by the hapten itself.

Furthermore, each mAb could at least recognize one within-group heterologous coating antigen; for example, mAb1B6 from FAM1 could bind FAM2-BSA and mAb4E10 could bind FAM5-BSA, as shown in Figure 4. The results indicate the maximal exposure of certain groups of haptens has an important effect on the profiles of the resultant antibody recognition [24]. Although all mAbs provided definite titers to coating antigens, only mAb5D11 from FAM5 showed the recognition ability to free FAM when pairing with the heterologous coating antigen FAM1-BSA, as shown in Figure 3C. The results of the coating antigen binding and free FAM recognizing suggest that the obtained mAbs, except 5D11, tightly bind not only to the hapten alone but also a fraction of the carrier protein to which the hapten has been linked.

### 3.4. mAb5D11 Characterization by ic-ELISA

A typical standard curve of ic-ELISA for the detection of FAM prepared with mAb5D11 and the coating FAM1-BSA is shown in Figure 3C. The calculated limit of detection (concentration of FAM corresponding to 10% specific binding, i.e., IC_10_) is 10 μg/mL with an IC_50_ value of 97 μg/mL in the buffer. Interestingly, mAb5D11 shows the recognition ability to FAM only when pairing with the heterologous coating antigen FAM1-BSA. This can be explained by the heterologous theory of competitive immunoassay. The competition strength between the antibody, analyte, and coating antigen is of primary importance in a competitive immunoassay. The heterology of coating hapten could result in a decrease in antibody recognition to the coating hapten compared with the immunizing hapten, thus allowing the analyte at lower concentrations to compete with the heterogeneous coating antigen [18].

In this study, as it can be observed in Figure 4, mAb5D11 could recognize all five coating haptens with varied binding ability expressed by the antibody dilution. It is not surprising that FAM4 and FAM5 used as coating haptens have a higher antibody dilution, meaning a stronger binding ability. The other three heterologous coating haptens showed lower antibody dilutions due to the heterology of both the linkage site and the spacer arm. As mentioned above, the relatively little binding ability of mAb5D11 to the heterologous coating hapten allows FAM to displace mAb5D11 from the antibody–antigen complex. Although not all heterologous coating antigens strictly follow the heterology theory, luckily, the heterologous FAM1 was successfully paired with mAb5D11 and could be potentially used to develop an immunoassay for the detection of FAM. The specificities of mAb5D11 were then evaluated with nine other structural/functional analogs including bromoacetic acid, chloroacetamide, chloroacetic acid, difluoroacetic acid, FAA, and chloroacetate, among others, showing that all of the CRs are below 0.1% (Table 1). The high specificity of mAb5D11 could guarantee the accuracy of the subsequently developed immunoassay.

## 4. Discussion

Hapten design is a crucial step to induce strong and specific antibody responses to the target of interest. Generally, an appropriate hapten should mimic the target molecule as much as possible in terms of structural resemblance, size, steric conformation, electronic configuration, and hydrophobic properties [25]. In the case of a small molecule like FAM, a rational spacer arm must be introduced to support a certain epitope away from the carrier protein and maximally expose it to the immune system [21,26]. The standard criterion for a spacer arm design is to introduce a linear aliphatic spacer arm to the target molecule [27]. For the FAM hapten design, a linear aliphatic spacer arm may be not enough to elicit a significant antibody response because of the infinitesimal and simple structure. Thus, the introduction of appropriately complicated and bulkier structures is expected, such as the phenyl group, which will act not only as a spacer arm but also assist in triggering the immune response by increasing the size of the resultant hapten. As observed in Table S1, in most of the small molecules, phenyl-contained spacer arms for hapten design were introduced, such as histamine and acrylamide [19,20].

In addition, the modification site of target molecules has also been shown to be important and needs to be considered [21]. In the case of FAM, the feature structure is fluorine, which should be preferentially exposed to the animal immune system. More importantly, the introduction of fluorine in the hapten design is reported to enhance immune recognition. Some studies have prepared fluorine-modified carbohydrates, peptides, and glycopeptides to boost the binding of T cell receptors, evoke a significant immune response, and enhance immune recognition [4,28,29]. One previous report showed that the fluorine-modified cocaine hapten elicited a higher antibody titer than the cocaine hapten because fluorine provides some distinct properties, such as the super hydrophobicity of fluorocarbons, inductive effect, and polar hydrophobicity [30]. Thus, we designed FAM1~FAM3 to maximally expose fluorine with different spacer arms as much as possible, which is expected to enhance the immune response. In contrast, FAM4 and FAM5 were designed to link the carbon adjacent to fluorine, exposing the amino group. A key point in the design of FAM haptens is the use of two kinds of spacer arms and two linking sites, providing a higher possibility to induce a specific antibody response and heterologous pairing of an immunizing hapten and coating hapten [31,32].

The study of immunization showed that only FAM5 successfully produced specific antibodies to FAM, indicating that a positive effect on the antibody response is not observed by increasing the exposure of fluorine compared with the amino group of haptens (FAM2 and FAM3). Furthermore, to evaluate the introduction of benzoylformic acid as a spacer arm on the effectiveness of the antibody response, we designed FAA haptens with benzoylformic acid as a spacer arm, and the results showed the FAA haptens could not induce specific antibodies to FAA. Thus, the phenyl-contained spacer arm incorporated in a hapten does not always guarantee the induction of a positive antibody response. Based on the antibody results from the haptens, we could conclude the group size of haptens may play an important role since the amino group of FAM5 is bigger than the fluorine of FAM3. The amino group theoretically could form more hydrogen bonds, which is one of the main molecular forces between the interaction of a hapten and antibody. More importantly, both exposing the amino group to an extreme and the involvement of benzoylformic acid as a spacer arm are necessary to produce antibodies to FAM by comparing FAM5 and FAM4. The importance of the amino group of FAM and benzoylformic acid as the spacer arm to induce a specific antibody to FAM could be confirmed.

The IC_50_ of ELISA based on mAb5D11 obtained was 97 μg/mL, which is comparable to those of antibodies to small molecules with a similar molecular weight, such as semicarbazide and acrylamide [19,33]. As we summarized in Table S1, the IC_50_ values of antibodies to molecules with a molecular weight below 150 Da significantly varied and generally are in the level of μg/mL. The molecules containing a heterocyclic ring are usually with a lower IC_50_ than the linear molecules like FAM. This is because the hydrophobic force may be the main driving force between a small molecule and the antibody [34].

The H binding may not be the main force between a hapten and antibody because, unlike protein antigens, small molecules have a limited potential for H bonds. This is why many molecules containing benzenes always could induce a good antibody regardless of the molecular weight of the hapten.

## 5. Conclusions

In summary, for the first time, the successful synthesis of FAM haptens and the production of highly specific mAbs to FAM are described. The obtained mAbs5D11 may be a potentially valuable reagent for the fast diagnosis of FAM poisoning and an antidote to treat patients in the clinic. In addition, the hapten design strategy of FAM provided useful clues for the production of specific antibodies against other small molecules.

## Figures and Tables

**Table 1:** The cross-reactivities (CRs) of mAb5D11 determined by icELISA.

Compounds	S/F ^1^	IC_50_ (μg/mL)	CR	Structure
FAM	S&F	100	100%	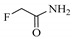
Bromoacetic acid	F	_	_	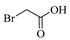
Chloroacetamide	F	_	_	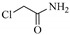
Chloroacetic acid	F	_	_	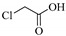
Difluoroacetic Acid	F	_	_	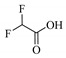
FAA	S&F	_	_	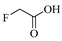
Thiosemicarbazide	F	_	_	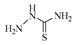
1-Chloro-3-fluoroisopropanol	F	_	_	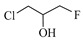
1,3-Difluoro-2-propanol	F	_	_	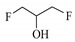
2,2,2-Trifluoroacetamide	F	_	_	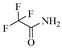

^1^ S represents structural analogs, F represents functional analogs.

## Supplementary Materials

The following are available online at https://www.mdpi.com/2218-273X/10/7/986/s1, Figure S1: the mass spectra and ^1^H NMR spectra of (A) FAM1, (B) FAM2, (C) FAM3, (D) FAM4, (E) FAM5, (F) FAA1, (G) FAA2, Scheme S1: the synthetic route of FAM1, Scheme S2: the synthetic route of FAM2, Scheme S3: the synthetic route of FAM3, Scheme S4: the synthetic route of FAM4, Scheme S5: the synthetic route of FAA1, Scheme S6: the synthetic route of FAA2, Table S1: Summary of reports about antibodies for very small molecules found in the literature and described in this study, Table S2: the detailed information of antibody titers and inhibition ratios of mice at the third immunizations, Table S3: the isotypes of mAbs.
