# Supplementary material for: Hapten Design and Monoclonal Antibody to Fluoroacetamide, a Small and Highly Toxic Chemical
Source: Biomolecules. 2020 Jul 1;10(7):986. doi: 10.3390/biom10070986 (PMC7407904; doi:10.3390/biom10070986)
Supplement: Supplementary file 1 [file biomolecules-10-00986-s001.pdf]

Supporting Information for Biomolecules

Hapten Design and Monoclonal Antibody to Fluoroacetamide, a Small and Highly Toxic Chemical

Ling Yang [1], Xiya Zhang [2], Dongshuai Shen [1], Xuezhi Yu [1], Yuan Li [1], Kai Wen [1], Jianzhong Shen [1] and Zhanhui Wang [1,*]

[1] Beijing Advanced Innovation Center for Food Nutrition and Human Health, College of Veterinary Medicine, China Agricultural University, Beijing Key Laboratory of Detection Technology for Animal-Derived Food Safety, and Beijing Laboratory for Food Quality and Safety, 100193 Beijing, PR China; yl16@cau.edu.cn (L.Y.); shends1992@cau.edu.cn (D.S.); yuxuezhi@cau.edu.cn (X.Y.); b20183050469@cau.edu.cn (Y.L.); wenkai@cau.edu.cn (K.W.); sjz@cau.edu.cn (J.S.)

[2] College of Food Science and Technology, Henan Agricultural University, 450002 Zhengzhou, PR China; zhangxiya@henau.edu.cn

* Correspondence: wangzhanhui@cau.edu.cn; Tel.: +86-10-6273 4565; Fax: +86-10-6273 1032

Table of Contents for Supporting Information

	Contents	Pages
1	Buffers	S4
2	Synthesis and characterization of haptens	S4–S24
3	Conjugates preparation and determination	S25
4	Immunization procedure of mice	S26
5	ELISA development and data analysis	S26–S27
6	Monoclonal antibody production	S27–S28

Supplementary Schemes, Tables and Figures

	Legends	Pages
Scheme S1	The synthetic route of FAM1	S4
Scheme S2	The synthetic route of FAM2	S5
Scheme S3	The synthetic route of FAM3	S7
Scheme S4	The synthetic route of FAM4	S9
Scheme S5	The synthetic route of FAA1	S12
Scheme S6	The synthetic route of FAA2	S16
Figure S1	The mass spectra and ^{1}H NMR spectra of (A) FAM1, (B) FAM2, (C) FAM3, (D) FAM4, (E) FAM5, (F) FAA1, (G) FAA2	S18–S24
Table S1	Summary of reports about antibodies for very small molecules found in the literature and described in the study	S29
Table S2	The detailed information of antibody titers and inhibition ratios of mice at the third immunizations	S31
Table S3	The isotypes of mAbs	S32

1. Buffers

The following buffers were used in the work: (1) coating buffer, 0.05 M sodium carbonate buffer, pH 9.6; (2) blocking buffer, 0.01 M phosphate-buffered saline (PBS), pH 7.4, 0.5% casein (w/v); (3) washing buffer, 0.01 M PBS with 0.05% Tween-20 (v/v); (4) antibody dilution buffer, 0.01 M PBS containing 0.2% albumin; (5) enzyme labeled secondary antibody dilution buffer, antibody dilution buffer containing 5% albumin (w/v); (6) substrate, 0.1% TMB (w/v) and H_2O_2 in 0.05 M citrate buffer, pH 4.5; and (7) stopping reagent, 2 M H_2SO_4.

2. Synthesis and characterization of haptens

2.1. Synthesis of FAM1

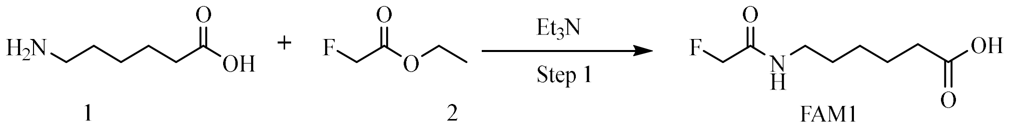

Scheme S1. The synthetic route of FAM1.

FAM1 was prepared according to the following scheme.

First, 6-aminocaproic acid (10.00 g, 76 mmol), triethylamine (Et_3N, 23.00 g, 227 mmol) and ethyl fluoroacetate (12.00 g, 113 mmol) in methanol (MeOH, 100 mL) were stirred at room temperature (RT) for 10 min, then refluxed for 24 h. The mixture was cooled to RT and adjusted pH to 5 with acetic acid and evaporated. The residue was re-dissolved in ethyl acetate (EtOAc) and washed by 30 mL of 0.9% NaCl, dried over Na_2SO_4, then filtered and evaporated to give the product FAM1 (7 g, 48%). HRMS (*m/z*) calc. for $C_8H_{14}FNO_3^{(-)}$ 191.10, found 190.08, ^{1}H NMR (400 MHz, $CDCl_3$) δ 6.48 (bs, 1H, NH), 4.87 (s, 1H, CH_2), 4.75 (s, 1H, CH_2), 3.35 (q, 2H, CH_2, J = 6.8 Hz), 2.36 (t, 2H, CH_2, J = 8.4 Hz), 1.67 (m, 2H, CH_2), 1.59 (m, 2H, CH_2), and 1.40 (m, 2H, CH_2).

2.2. Synthesis of FAM2

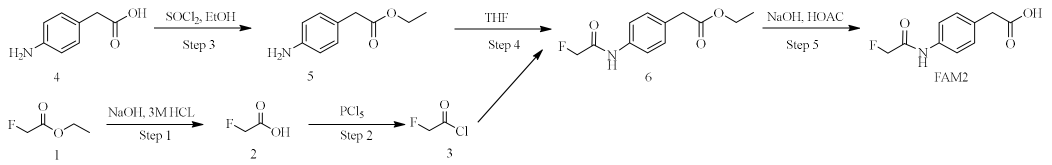

Scheme S2. The synthetic route of FAM2.

FAM2 was prepared according to the following scheme in 5 steps.

Step 1 Synthesis of compound 2 (2-fluoroacetic acid)

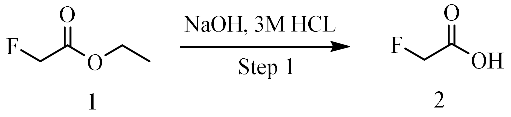

A mixture of NaOH (13.50 g, 339 mmol) and compound 1 (30.00 g, 283 mmol) in ethanol (300 mL) was stirred at RT for 24 h; then the mixture was evaporated by rotary evaporation. After that, the residue was subject to 3 M HCl (200 mL), extracted with ether (4 × 50 mL), filtered, evaporated, dried over Na_2SO_4, and obtained the crude solid compound 2 (15.00 g).

Step 2 Synthesis of compound 3 (2-fluoroacetyl chloride)

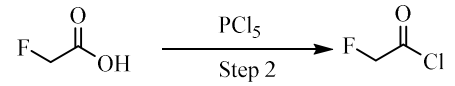

2 3

Table 5. 44.00 g, 211 mmol) was added to a three-neck round-bottom flask and cooled to 0°C, and the compound 2 (15.00 g) was slowly added and stirred at RT for 0.5 h. Then the mixture was heated in an oil bath until the effluent came out at 80°C for 1 h. The distillate at 70–72°C was collected to give a yield of 12.00 g of a colorless compound 3 (44%).

Step 3 Synthesis of compound 5 (ethyl 2-(4-aminophenyl)acetate).

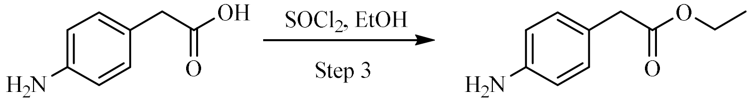

4 5

The mixture of thionyl chloride ($SOCl_2$, 17.3 g, 146 mmol) and compound 4 (11.00 g, 72.8 mmol) in ethanol (90 mL) was stirred at RT for 1 h. The mixture was adjusted pH to 8~9 with Na_2CO_3 and extracted with ethyl acetate (EtOAc, 3 × 100 mL). The combined extract was washed by 30 mL of 0.9% NaCl, dried over Na_2SO_4, filtered, and evaporated to give light-yellow liquid compound 5 (13.00 g, 100%).

Step 4 Synthesis of compound 6 (ethyl 2-(4-(2-fluoroacetamido)phenyl)acetate)

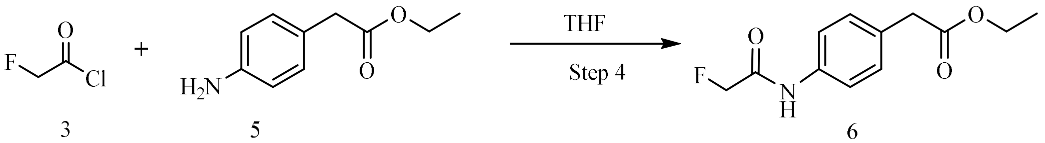

3 5 6

The solution of compound 3 (12.00 g, 44%) and compound 5 (13.00 g, 100%) in tetrahydrofuran (THF, 100 mL) was stirred at RT for 2 h. The combined organic layers were extracted with EtOAc (3 × 100 mL), washed by 30 mL of 0.9% NaCl, dried over Na_2SO_4. The residue was purified by silica gel chromatography separation (DCM: MeOH = 80: 1) to give a yellow solid compound 6 (12.50 g, 72%).

Step 5 Synthesis of FAM2

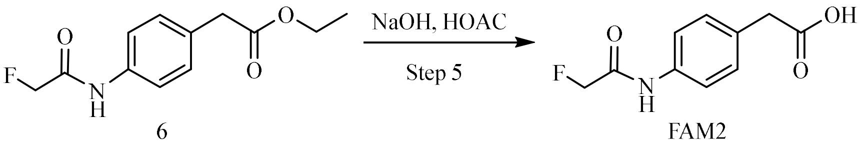

6 FAM2

After that, NaOH (1.80 g, 45 mmol) in water (25 mL) was added in compound 6 (12.50 g, 72%) dissolved in ethanol (70 mL), stirred at RT for 1 h, and the pH was adjusted to 5 with acetic acid followed by filtering, washing, and drying steps. The filter residue was dried, and a yellow powder was obtained (3.70 g, 59%) FAM2, HRMS (*m*/*z*) calc. for $C_{10}H_{10}FNO_3$ $^{(+)}$ 211.06, found 212.07, ^1H NMR (DMSO-d_6, 400 MHz) δ 3.52 (s, 2H), 4.92 (s, 1H), 5.03 (s, 1H), 7.21 (d, *J* = 4.2 Hz, 2H), 7.57 (d, *J* = 4.2 Hz, 2H), and 10.07 (br, 1H).

2.3. Synthesis of FAM3

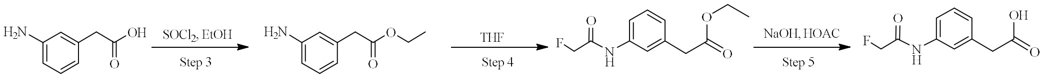

4 5 6 FAM 3

Scheme S3. The synthetic route of FAM3.

FAM3 was prepared according to the following steps. The first two steps were already described in the procedure of FAM2 preparation.

Step 3 Synthesis of compound 5 (ethyl 2-(3-aminophenyl)acetate).

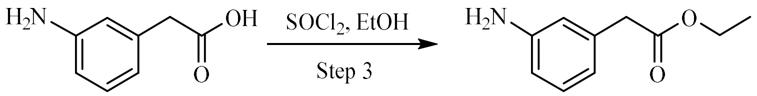

4 5

The mixture of thionyl chloride ($SOCl_2$, 15.70 g, 132 mmol) and compound 4 (10.00 g, 66 mmol) in ethanol (80 mL) was stirred at RT for 1 h. The mixture was adjusted pH to 8–9 with Na_2CO_3 and extracted with EtOAc (3 × 100 mL). The combined extract was washed by 30 mL of 0.9% NaCl, dried over Na_2SO_4, filtered, and evaporated to give yellow liquid compound 5 (11.86 g, 100%).

Step 4 Synthesis of compound 6 (ethyl 2-(3-(2-fluoroacetamido)phenyl)acetate)

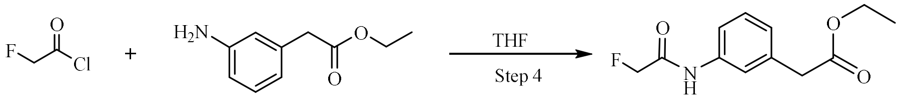

3 5 6

Next, the solution of compound 3 (1.91 g, 20 mmol) and compound 5 (3.14 g) in tetrahydrofuran (THF, 30 mL) was stirred at RT for 2 h. The combined organic layers were extracted with EtOAc (3 × 100 mL), washed by 30 mL of 0.9% NaCl, dried over Na_2SO_4. The residue was purified by silica gel chromatography separation (PE: EA = 1: 3) to give a yellow solid compound 6 (2.73 g, 12 mmol, 65%).

Step 5 Synthesis of FAM3

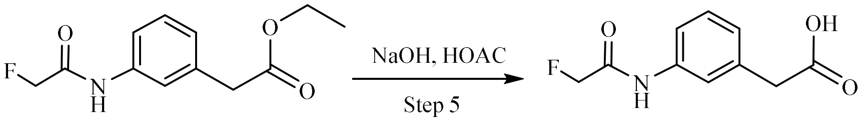

6 FAM3

After that, NaOH (1.80 g, 45 mmol) in water (25 mL) was added in compound 6 (2.73 g, 12 mmol, 65%) dissolved in ethanol (30 mL), stirred at RT for 1 h, and the pH was adjusted to 5 with acetic acid followed by filtering, washing, and drying steps. The filter residue was dried and a white powder of FAM3 was obtained (1.29 g, 7 mmol, 53%), HRMS (*m*/*z*) calc. for $C_{10}H_{10}FNO_3$ $^{(+)}$ 211.06, found 212.07, ^{1}H NMR (400 MHz, DMSO-d_6) δ 12.31 (s, 1H, -COOH), 10.04 (s, 1H, -CONH), 7.53 (m, 2H, ArH), 7.22 (m, 1H, ArH), 6.07 (d, *J* = 7.6 Hz, 1H, ArH), 4.95 (d, *J* = 46.8 Hz, 2H, -FCH_2), and 3.52 (s, 2H, CH_2).

2.4. Synthesis of FAM4

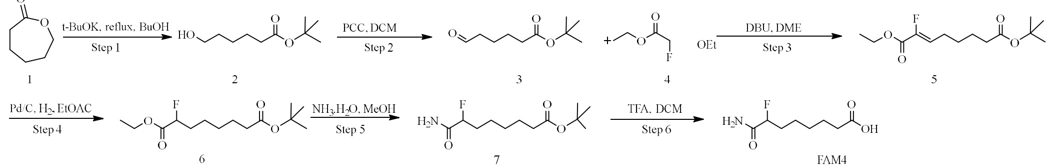

Scheme S4. The synthetic route of FAM4.

FAM4 was prepared according to the following scheme in 6 steps.

Step 1 Synthesis of compound 2 (*tert*-butyl 6-hydroxyhexanoate)

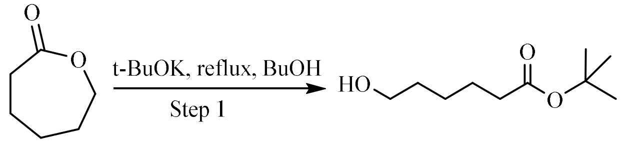

1 2

A mixture of compound 1 (10.96 g, 96 mmol) and t-BuOK (21.54 g, 192 mmol) in t-BuOH (300 mL) was heated under reflux for 8 h. The reaction mixture was cooled to RT and poured onto water (1000 mL). The mixture was extracted with toluene (2 × 500 mL). The combined organic layers were washed with water (2 × 1000 mL), brine (500 mL), dried over Na_2SO_4. Solvent removal gave a yellow oil (5.70 g, 32%) as a crude product.

Step 2 Synthesis of compound 3 (*tert*-butyl 6-oxohexanoate)

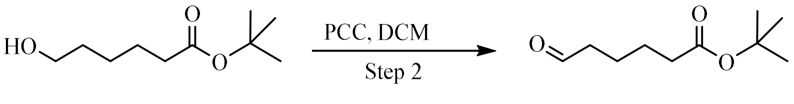

2 3

To a solution of compound 2 (5.70 g, 30.32 mmol) in DCM (100 mL) cooled to 0°C was added PCC (7.20 g, 33.4 mmol). The reaction mixture was then stirred at RT for 4 h. Celite (20.00 g) was added to the reaction mixture. The mixture was filtered and concentrated to give a residue. The residue was further purified by silica gel chromatography (silica gel, PE: EtOAc = 10: 1) to give a colorless oil (3.60 g, 59%) as compound 3.

Step 3 Synthesis of compound 5 (8-(*tert*-butyl) 1-ethyl (Z)-2-fluorooct-2-enedioate)

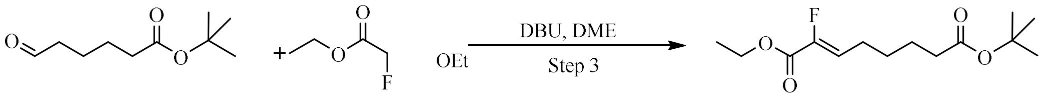

3 4 5

To a solution of compound 3 (3.60 g, 18 mmol) and compound 4 (6.54 g, 27 mmol) in THF (50 mL) was added DBU (4.10 g, 27 mmol). The resulting mixture was stirred at RT for 1 h. Saturated ammonium chloride (100 mL) was added to the reaction mixture and extracted with EtOAc (2 × 150 mL). The combined organic layers were washed with water (200 mL) and then brine (200 mL), and then they were dried over Na_2SO_4. Solvent removal gave a residue. The residue was purified with silica gel chromatography (PE: EtOAc = 10: 1) to afford a colorless oil (3.20 g, 65%) as compound 5.

Step 4 Synthesis of compound 6 (8-(*tert*-butyl) 1-ethyl 2-fluorooctanedioate)

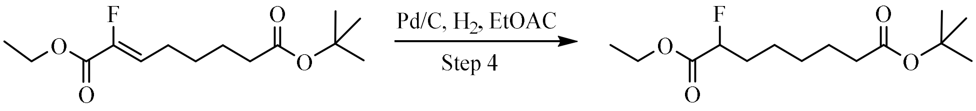

5 6

To a solution of compound 5 (3.20 g, 11.7 mmol) in EtOAc (30 mL) was added palladium on carbon (10%, 0.30 g). The mixture was hydrogenated under a H_2 balloon for 16 h. The mixture was filtered to remove palladium on carbon. The filtrate was concentrated to afford a pale-yellow oil as compound 6 (2.90 g, 91%).

Step 5 Synthesis of compound 7 (*tert*-butyl 8-amino-7-fluoro-8-oxooctanoate)

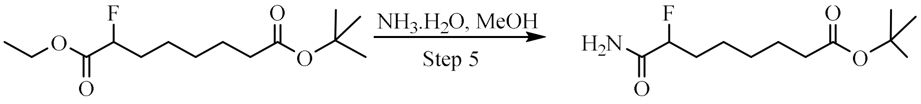

6 7

To a solution of compound 6 (5.88 g, 21 mmol) in MeOH (50 mL) was added ammonia (28% in water (5.88 g, 168 mmol)). The mixture was heated at 50°C for 16 h. The mixture was concentrated under vacuum to give compound 7 (4.12 g, 79%).

Step 6 Synthesis of FAM4

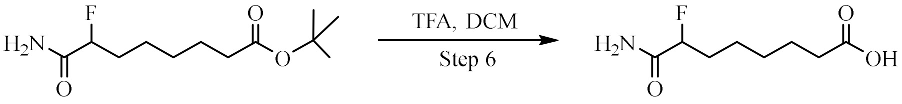

7 FAM4

TFA (8 mL) was added to a solution of compound **7** (4.12 g, 16.6 mmol) in DCM (40 mL). The mixture was stirred at RT for 16 h. The reaction mixture was then concentrated. To the reaction mixture was added saturated $NaHCO_3$ to adjust pH to 9. To the reaction mixture was then added 6 M HCl to adjust pH to 1. A white precipitate appeared, which was collected via filtration. The solid was dried under vacuum to give FAM4 (1.92 g, 61%). HRMS (*m*/*z*) calc. for $C_8H_{14}FNO_3{}^{(-)}$ 191.10, found 190.08, ^1H NMR (500 MHz, DMSO-d_6) δ 1.28–1.37 (m, 4H), 1.48–1.51 (m, 2H), 1.67–1.81 (m, 2H), 2.19 (t, *J* = 7.5 Hz, 2H), 4.74–4.86 (m, 1H), 7.39 (s, br, 1H), 7.54 (s, br, 1H), and 11.98 (s, 1H).

2.5. Synthesis of FAA1

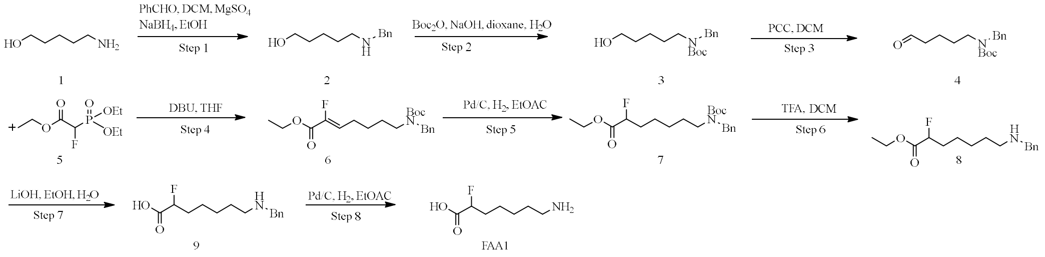

Scheme S6. The synthetic route of FAA1.

FAA1 was prepared according to the following scheme in 8 steps.

Step 1 Synthesis of compound 2 (5-(benzylamino)pentan-1-ol)

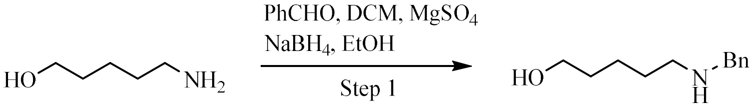

1 2

To a solution of compound 1 (23.90 g, 231.67 mmol) in DCM (400 mL) was added benzaldehyde (29.50 g, 278 mmol), followed by the addition of anhydrous magnesium sulfate (70 g). The reaction mixture was stirred at RT for 16 h. The reaction mixture was filtered to remove the solid. The filtrate was concentrated under vacuum. To the residue was added EtOH (500 mL), followed by the addition of NaBH4 (29.12 g, 462 mmol). The mixture was stirred at RT for 4 h. The organic solvent was removed under vacuum. To the residue was added DCM (500 mL). The mixture was washed with water (2 × 500 mL), dried over Na_2SO_4, and concentrated to give an oily residue (32.43 g). The residue was dissolved in DCM (200mL). To the mixture was added 1M HCl (150 mL) and the mixture was stirred for 30 minutes. The organic layer was separated. The aqueous layer was treated with 1 N

NaOH to adjust pH to 10. The mixture was extracted with DCM (2 × 150 mL), the combined extracts were dried over anhydrous Na_2SO_4 and concentrated to give a light-yellow oil as compound 2 (22.32 g, 45%).

***Step* 2** Synthesis of compound 3 (*tert*-butyl benzyl(5-hydroxypentyl)carbamate)

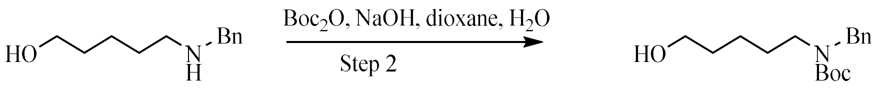

2 3

Table 2. (21.30 g, 110.6 mmol) in dioxane (100 mL) was added NaOH solution (21.30 g, 221.2 mmol in 100 mL of water), followed by the addition of Boc_2O (36.30 g, 166 mmol). The resulting mixture was stirred at RT for 16 h. To the mixture was added 1 N HCl (350 mL). The mixture was extracted with DCM (2 × 150 mL). The combined organic extracts were washed with 1 M HCl (2 × 100 mL), dried over Na_2SO_4. Concentration under vacuum gave an oily residue. The residue was subjected to silica gel column chromatography separation (PE: EtOAc = 5: 1 to 1: 1) to give a colorless oil as compound 3 (27.10 g, 84%).

***Step 3* Synthesis of compound 4 (*tert*-butyl benzyl(5-oxopentyl)carbamate).**

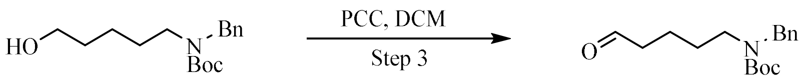

3 4

Silica gel (45 g) was added to a solution of compound 3 (27.10 g, 92 mmol) in DCM (300 mL), followed by the addition of PCC (30.00 g, 138 mmol). The mixture was stirred at RT for 16 h. The mixture was concentrated and subjected to silica gel column chromatography separation (PE: EtOAc = 10: 1 to 4: 1) to afford a colorless oil (19.80 g, 75%) as compound 4.

Step 4 Synthesis of compound 6 (ethyl (Z)-7-(benzyl(*tert*-butoxycarbonyl)amino)-2-fluorohept-2-enoate)

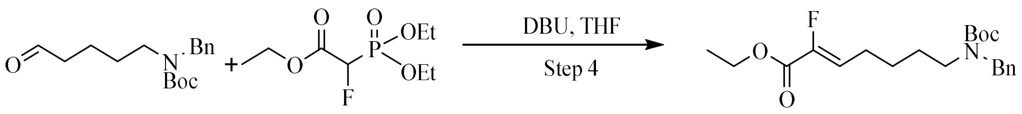

4 5 6

DBU (15.60 g, 64.4 mmol) was added to a solution of compound 4 (15.00 g, 51.5 mmol) and compound 5 (9.80 g, 64.4 mmol) in THF (150 mL). The mixture was stirred at RT for 16 h. Then, 1 M HCl (150 mL) was added to the mixture. The mixture was extracted with EtOAc (2 × 150 mL). The combined organic layers were washed with brine (150 mL), dried over Na_2SO_4, and concentrated. The residue was separated with silica gel column (PE: EtOAc = 10: 1) to give a colorless oil (13.72 g, 70%) as compound 6 (cis and trans mixture of double bond, ratio 1 to 1).

Step 5 Synthesis of compound 7 (ethyl 7-(benzyl(*tert*-butoxycarbonyl)amino)-2-fluoroheptanoate)

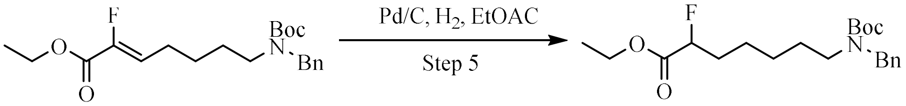

6 7

Palladium on carbon (10%, 2.00 g) was added to a solution of compound 6 (13.72 g, 36.2 mmol) in EtOAc (150 mL). The resulting mixture was hydrogenated under a hydrogen balloon for 18 h.

Palladium on carbon was removed with filtration. The filtrate was concentrated under vacuum to give a colorless oil (12.78 g, 92%) as compound 7.

Step 6 Synthesis of compound 8 (ethyl 7-(benzylamino)-2-fluoroheptanoate)

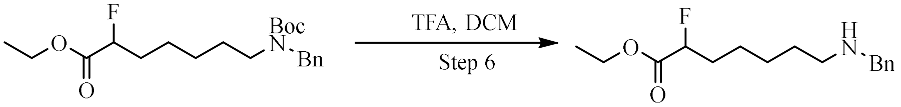

7 8

Table 30. mL) was added to a solution of compound 7 (11.87 g, 31.2 mmol) in DCM (100 mL). The mixture was stirred at RT for 16 h. The mixture was concentrated under vacuum to give a residue. The residue was dissolved in DCM (100 mL) and washed with saturated $NaHCO_3$ (100 mL), dried over Na_2SO_4. The mixture was concentrated under vacuum to give a residue (8.20 g, 94%) as compound 8.

Step 7 Synthesis of compound 9 (7-(benzylamino)-2-fluoroheptanoic acid)

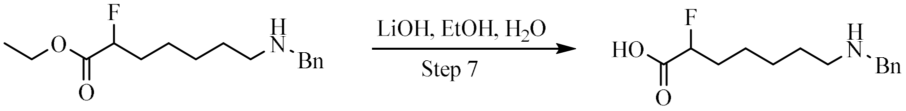

8 9

LiOH (2.52 g, 60 mmol) was added to a mixture of compound 8 (5.62 g, 20 mmol) in MeOH (30 mL) and water (30 mL). The mixture was then stirred at RT for 16 h. The mixture was concentrated. Water (20 mL) was added to the residue. The pH value of the mixture was adjusted to 4 with the addition of 1 N HCl. The white precipitate was collected by filtration and dried to afford a white solid (4.30 g, 85%) as compound 9.

Step 8 Synthesis of FAA1

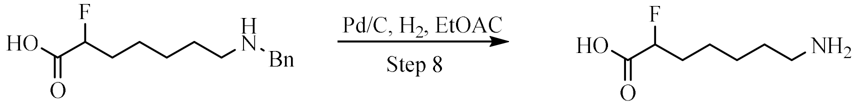

9 FAA1

To a mixture of compound 9 (4.30 g, 17 mmol) in MeOH (15 mL) and water (20 mL) was added palladium on carbon (10%, 2.00 g). The mixture was hydrogenated at 25°C under a hydrogen gas balloon for 96 h. The mixture was filtered to remove palladium on carbon. The filtrate was concentrated. Water was added to the residue to obtain a solution. The mixture was washed with DCM (2 × 20 mL). The aqueous layer was concentrated under vacuum to give a white solid (1.50 g, 56%) as FAA1. HRMS (*m*/*z*) calc. for $C_7H_{14}FNO_2$ $^{(+)}$ 163.10, found 164.11, ^{1}H NMR (300 MHz, DMSO-*d*$_6$) δ 1.33 (m, 4H), 1.57–1.75 (m, 4H), 2.72 (m, 2H), 4.51–4.68 (m, 1H), and 8.49 (s, br, 1H).

2.7. Synthesis of FAA2

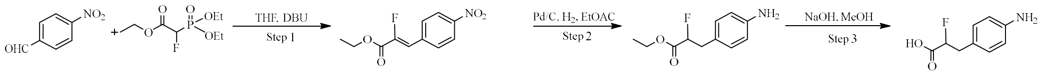

1 2 3 4 FAA2

Scheme S7. The synthetic route of FAA2.

FAA2 was prepared based on the following scheme in 3 steps.

Step 1 Synthesis of compound 3 (ethyl (Z)-2-fluoro-3-(4-nitrophenyl)acrylate)

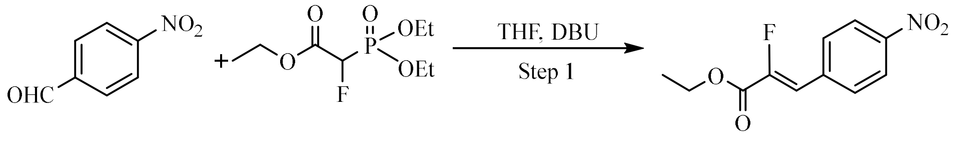

1 2 3

DBU (6.08 g, 40 mmol) was added to a solution of compound 1 (4.00 g, 26.47 mmol) and compound 2 (9.61 g, 39.7 mmol) in THF (70 mL). The mixture was stirred at RT for 2 h. To the reaction mixture was added 1 N HCl (30 mL). The mixture was extracted with EtOAc (3 × 50 mL). The extracts were combined and dried over Na_2SO4. The solvent was removed, the residue was subject to silica gel column chromatography (PE: EtOAc = 20: 1) to give a yellow powder (4.30 g, 68%) as compound 3.

Step 2 Synthesis of compound 4 (ethyl 3-(4-aminophenyl)-2-fluoropropanoate)

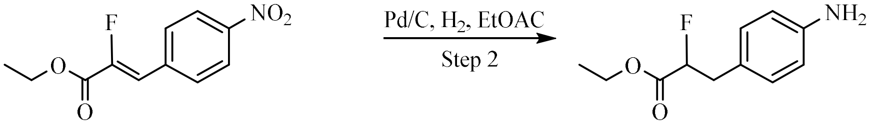

3 4

Palladium (10%, 1.00 g) was added to a solution of compound 3 (4.30 g, 18 mmol) in EtOAc (120 mL). The mixture was hydrogenated under a hydrogen balloon for 16 h. The mixture was filtered to remove palladium on carbon. The filtrate was concentrated. The residue was purified with silica gel column (PE: EtOAc = 20: 1) to give a red oil (1.90 g, 50%) as compound 4.

Step 3 Synthesis of FAA2

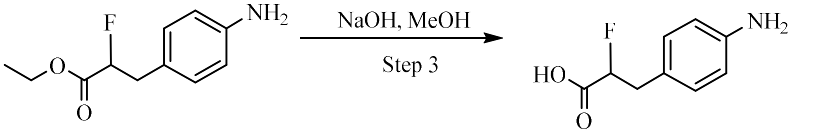

4 FAA2

A solution of NaOH (1.10 g, 27.1 mmol in 10 mL of water) was added to a suspension of compound 4 (2.86 g, 13.55 mmol) in MeOH (30 mL). The reaction mixture was stirred for 16 h at RT. The pH value of the reaction mixture was adjusted to 4 with 2 M HCl. The precipitate was filtered and dried in vacuum to give FAA2 (2.00 g, 81%). HRMS (*m/z*) calc. for $C_9H_{10}FNO_2$ $^{(+)}$ 183.07, found 184.07, ^{1}H NMR (300 MHz, DMSO-d_6) δ 2.78–3.07 (m, 2H), 4.96–5.16 (m, 1H), 6.49 (d, J = 8.1 Hz, 2H), 6.89 (d, J = 8.1 Hz, 2H), and 7.45 (s, br, 2H).

A

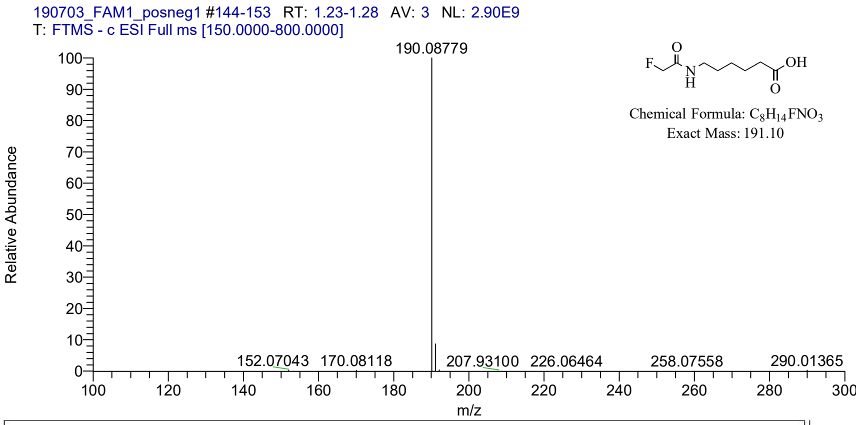

190703_FAM1_posneg1 #144-153 RT: 1.23-1.28 AV: 3 NL: 2.90E9
T: FTMS - c ESI Full ms [150.0000-800.0000]
190.08779
152.07043
170.08118
207.93100
226.06464
258.07558
290.01365
Relative Abundance
m/z
Chemical Formula: C8H14FNO3
Exact Mass: 191.10

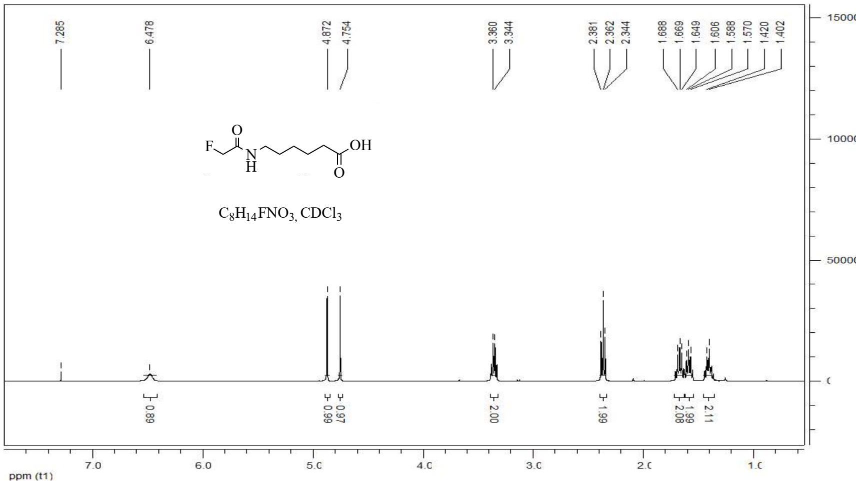

7.285
6.478
4.872
4.754
3.360
3.344
2.381
2.362
2.344
1.688
1.669
1.649
1.606
1.588
1.570
1.420
1.402
C8H14FNO3, CDCl3
0.89
0.99
0.97
2.00
1.99
2.08
1.99
2.11
ppm (t1)

B

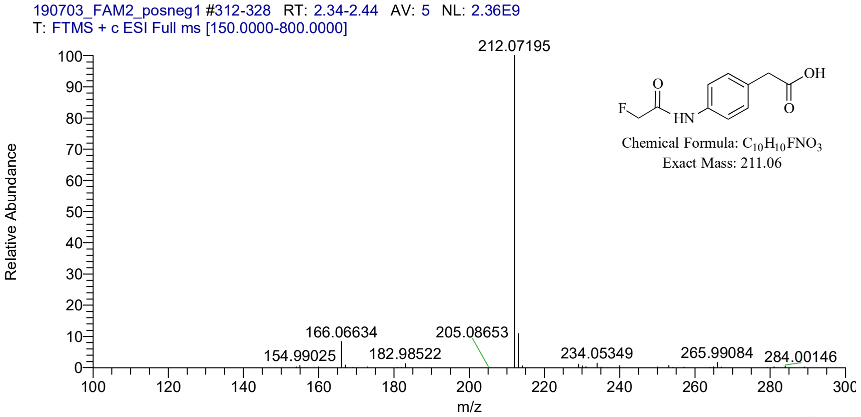
190703_FAM2_posneg1 #312-328 RT: 2.34-2.44 AV: 5 NL: 2.36E9
T: FTMS + c ESI Full ms [150.0000-800.0000]
212.07195
166.06634
205.08653
154.99025
182.98522
234.05349
265.99084
284.00146
Relative Abundance
m/z
Chemical Formula: C10H10FNO3
Exact Mass: 211.06

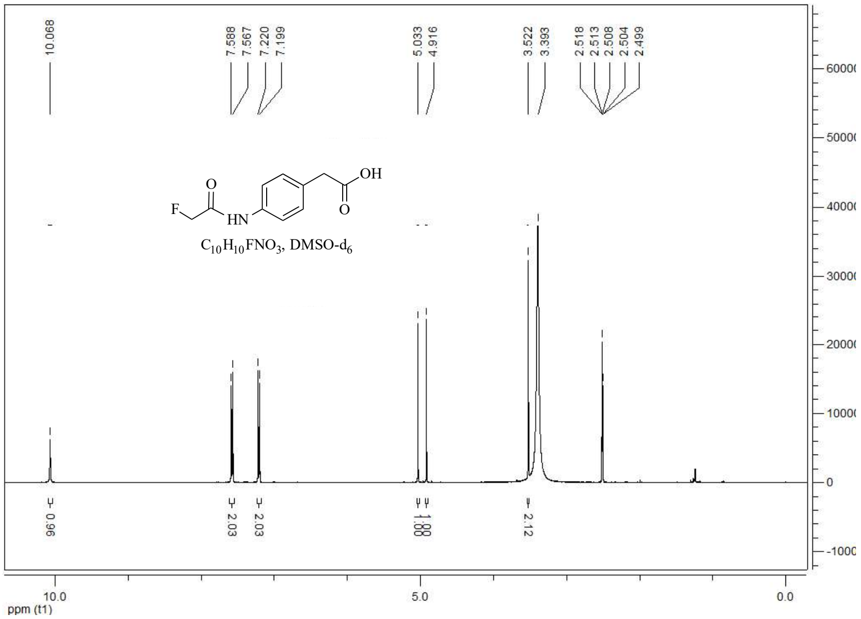
10.068
7.588
7.567
7.220
7.199
5.033
4.916
3.522
3.393
2.518
2.513
2.508
2.504
2.499
C10H10FNO3, DMSO-d6
0.96
2.03
2.03
1.88
2.12
ppm (t1)

C

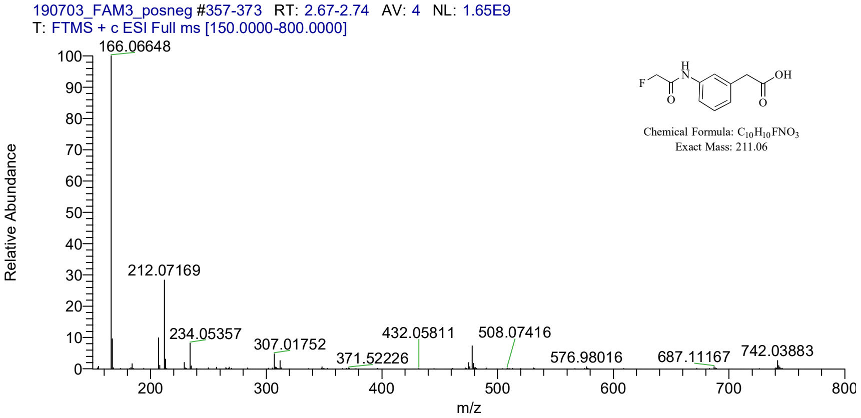
190703_FAM3_posneg #357-373 RT: 2.67-2.74 AV: 4 NL: 1.65E9
T: FTMS + c ESI Full ms [150.0000-800.0000]
166.06648
212.07169
234.05357
307.01752
371.52226
432.05811
508.07416
576.98016
687.11167
742.03883
Relative Abundance
m/z
Chemical Formula: C10H10FNO3
Exact Mass: 211.06

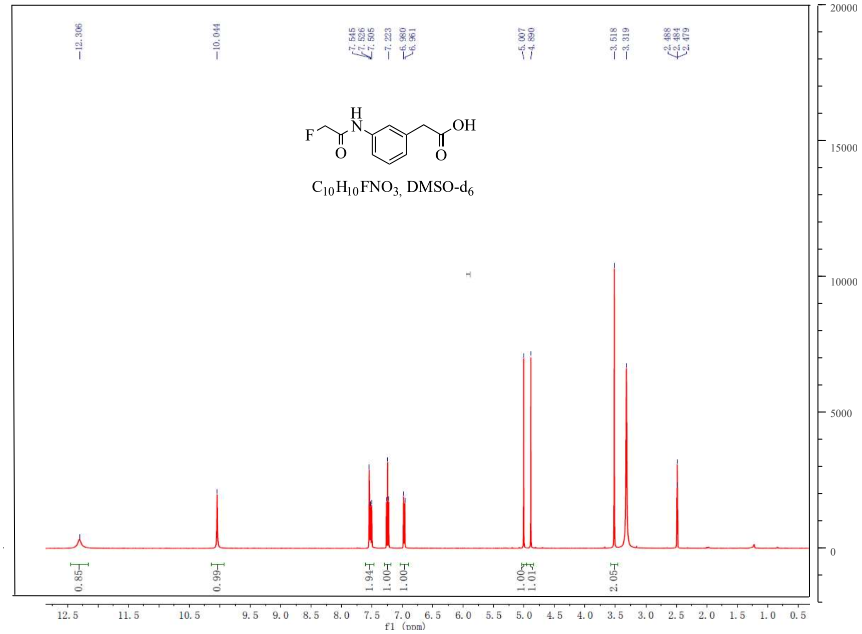
C10H10FNO3, DMSO-d6
12.306
10.044
7.545
7.526
7.505
7.223
6.980
6.961
5.007
4.890
3.518
3.319
2.488
2.484
2.479
0.85
0.99
1.94
1.00
1.00
1.00
1.01
2.05
f1 (ppm)

D

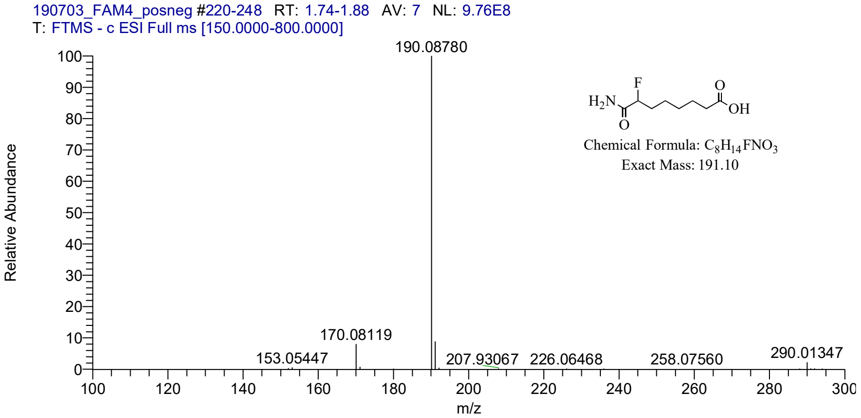
190703_FAM4_posneg #220-248 RT: 1.74-1.88 AV: 7 NL: 9.76E8
T: FTMS - c ESI Full ms [150.0000-800.0000]
190.08780
170.08119
153.05447
207.93067
226.06468
258.07560
290.01347
Relative Abundance
m/z
Chemical Formula: $C_8H_{14}FNO_3$
Exact Mass: 191.10

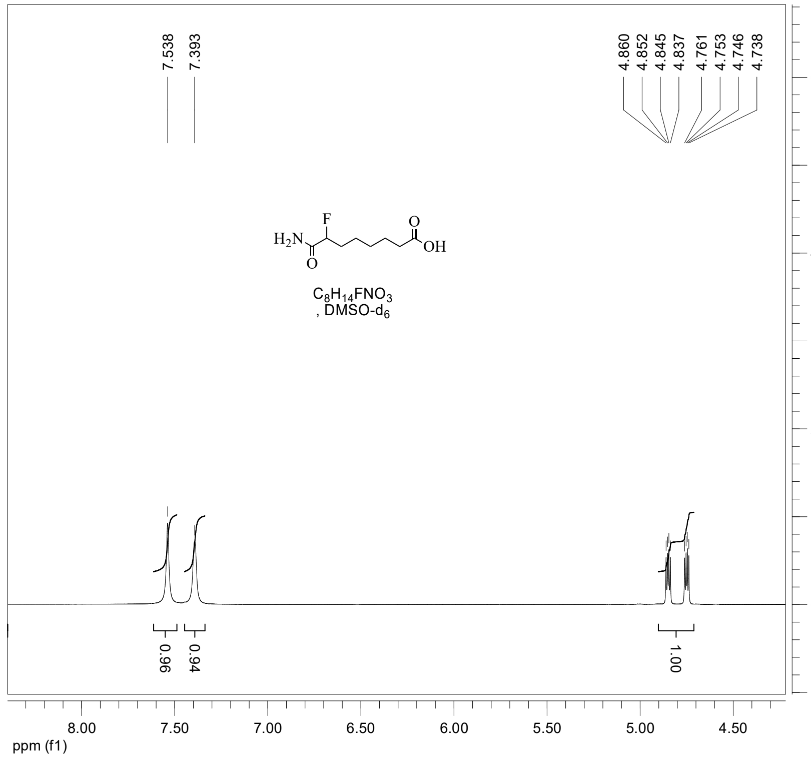
7.538
7.393
4.860
4.852
4.845
4.837
4.761
4.753
4.746
4.738
$C_8H_{14}FNO_3$
, DMSO-d_6
0.96
0.94
1.00
ppm (f1)

E

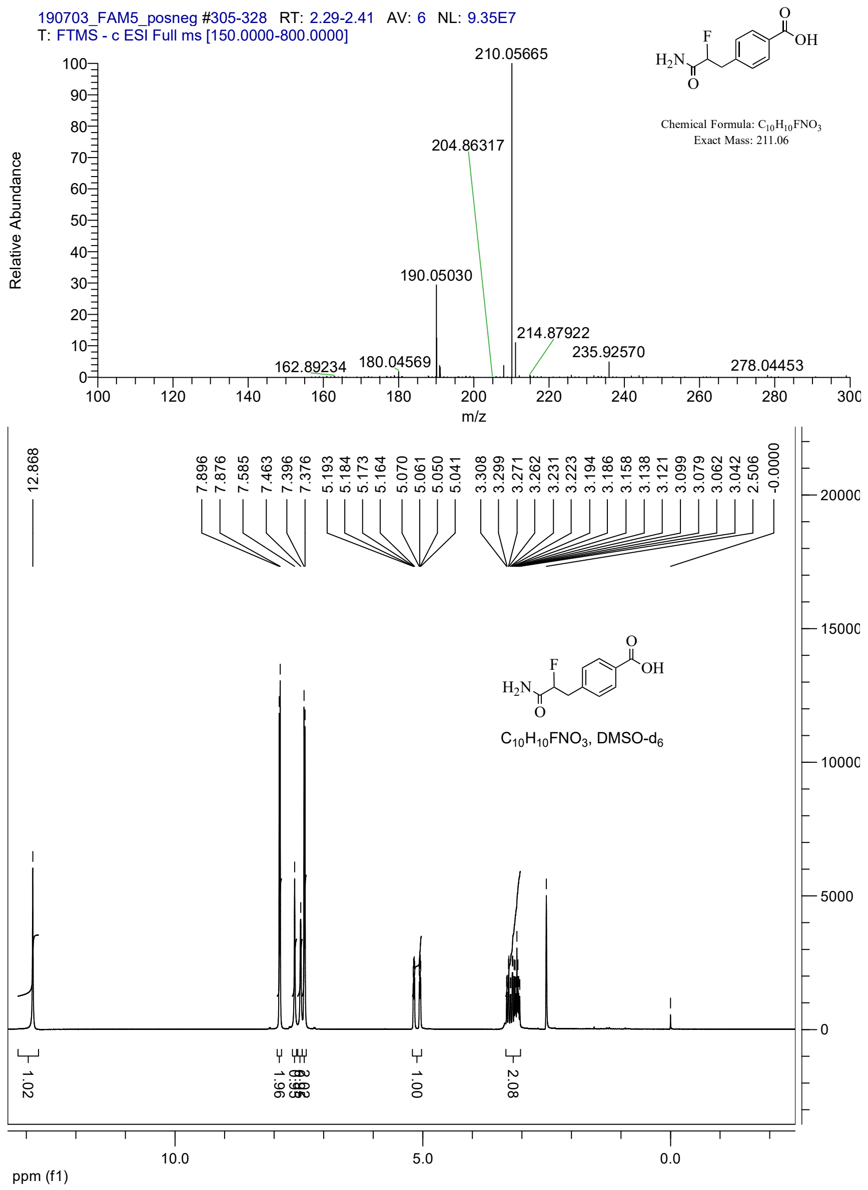

190703_FAM5_posneg #305-328 RT: 2.29-2.41 AV: 6 NL: 9.35E7
T: FTMS - c ESI Full ms [150.0000-800.0000]
210.05665
204.86317
190.05030
214.87922
235.92570
162.89234
180.04569
278.04453
Relative Abundance
m/z
Chemical Formula: C10H10FNO3
Exact Mass: 211.06
12.868
7.896 7.876 7.585 7.463 7.396 7.376 5.193 5.184 5.173 5.164 5.070 5.061 5.050 5.041 3.308 3.299 3.271 3.262 3.231 3.223 3.194 3.186 3.158 3.138 3.121 3.099 3.079 3.062 3.042 2.506 -0.0000
C10H10FNO3, DMSO-d6
1.02
1.96
1.00
2.08
ppm (f1)

F

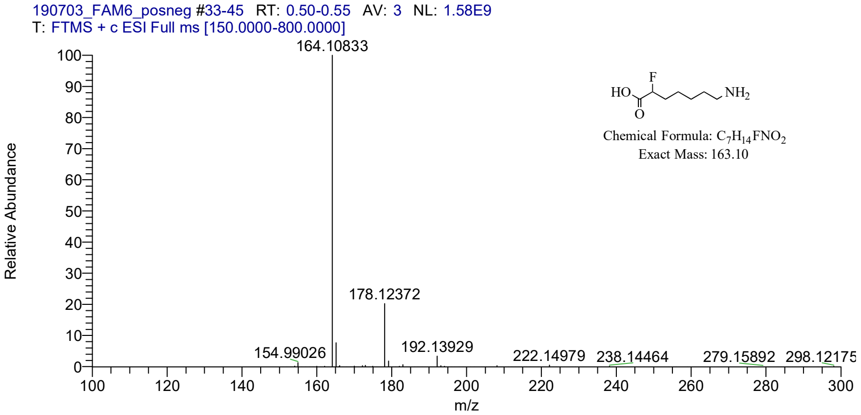
190703_FAM6_posneg #33-45 RT: 0.50-0.55 AV: 3 NL: 1.58E9
T: FTMS + c ESI Full ms [150.0000-800.0000]
164.10833
178.12372
192.13929
154.99026
222.14979
238.14464
279.15892
298.12175
Relative Abundance
m/z
Chemical Formula: C7H14FNO2
Exact Mass: 163.10

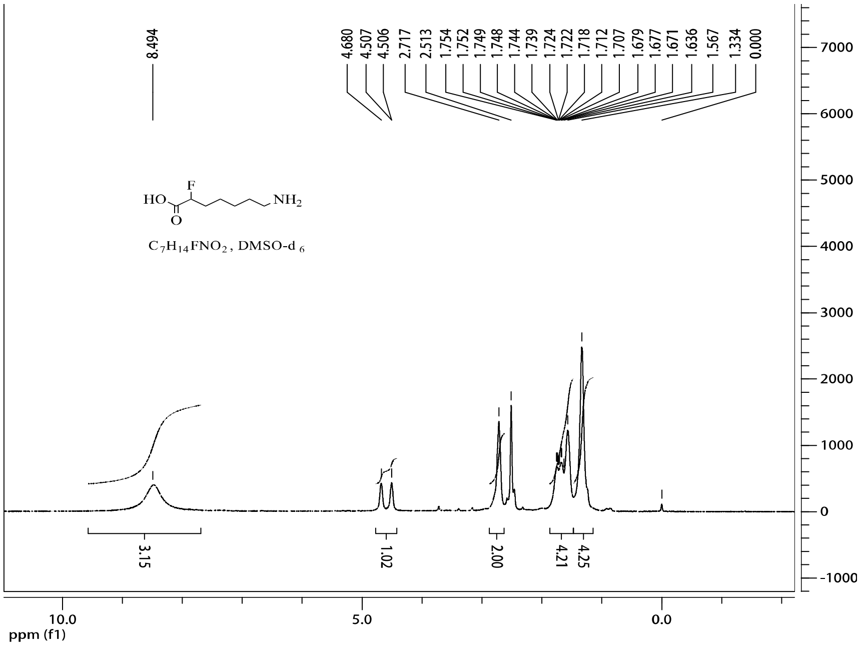
8.494
4.680
4.507
4.506
2.717
2.513
1.754
1.752
1.749
1.748
1.744
1.739
1.724
1.722
1.718
1.712
1.707
1.679
1.677
1.671
1.636
1.567
1.334
0.000
C7H14FNO2, DMSO-d6
3.15
1.02
2.00
4.21
4.25
ppm (f1)

G

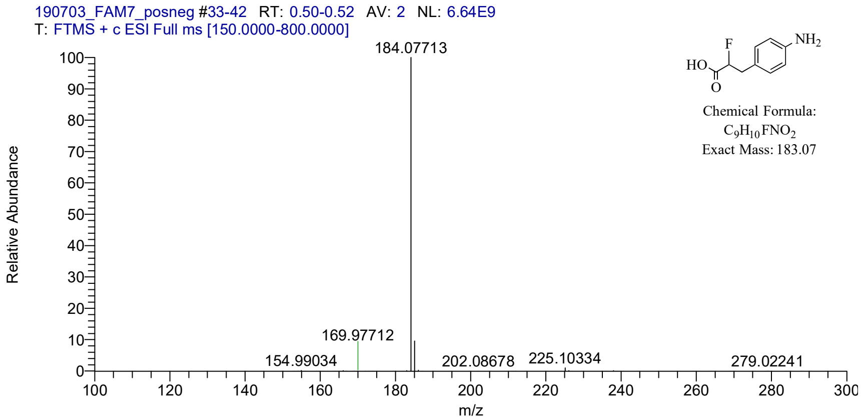

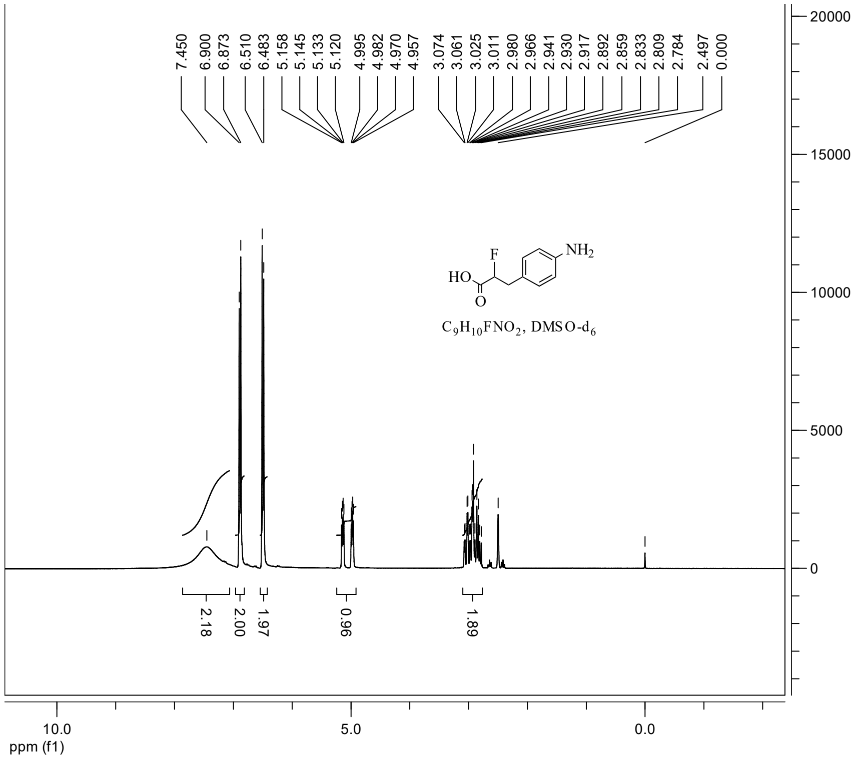

Figure S1. The mass spectra and ^{1}H NMR spectra of (**A**) FAM1, (**B**) FAM2, (**C**) FAM3, (**D**) FAM4, (**E**) FAM5, (**F**) FAA1, and (**G**) FAA2.

3. Conjugates preparation and determination

3.1. Preparation of FAM1-FAM5 conjugates

Active ester method was used for the preparation of immunogens hapten-KLH and coating antigens hapten-BSA. Briefly, hapten (40 mg), DCC (80 mg), and NHS (80 mg) were sequentially added in 3 mL of DMF, and the mixture was kept magnetically stirred (400 rpm) at RT for 6 h in darkness. The activated haptens were added drop-wise to a 10 mL solution of 1 mg mL^{-1} KLH or 10 mg mL^{-1} BSA in PBS (pH 7.4). The reactive solution was kept stirred for another 12 h at RT, dialyzed against PBS for 3 d, and then stored at –20°C until use.

3.2. Preparation of FAA1 and FAA2 conjugates

The immunogens hapten-cKLH and the coating antigens hapten-cBSA were prepared by activated carboxylic acids of carrier proteins with carbodiimide modification methods. In the first step, EDC was added to a final concentration of 2 mg/mL to a solution of 1 mg mL^{-1} KLH or 10 mg mL^{-1} BSA in 0.1 M PBS (pH 7.4), followed by magnetically stirred (400 rpm) at RT for 2 h. Subsequently, the solution was dialyzed against PBS for 3 d. Activated KLH and BSA carrier proteins were defined as cKLH and cBSA. In the second step, each hapten was coupled to cKLH and cBSA through its aminol group. To each carrier, 15 mM hapten was added. The solution was kept stirred for 2 h at RT, followed by dialysis against PBS for 3 d, and stored at –20°C.

The all prepared BSA conjugates were identified using MALDI-TOF/MS and were calculated as follows:

$$Conjugation\,ratio\ = \frac{\{M\,(conjugates) - M\,(BSA)\}}{M}(haptens) \qquad (1)$$

4. Immunization procedure of mice

Eight female BALB/c mice (eight weeks old) were immunized subcutaneously with 1: 1 emulsified mixture (*v/v*, 200 μL) of Hapten-KLH conjugates (100 μg) in PBS and Freund's complete adjuvant for the primary immunization and Freund's incomplete adjuvant for every four weeks booster immunization. One week after each booster injection, antibody titers and affinity were determined by non-competitive ELISA (nc-ELISA) and indirect competitive ELISA (ic-ELISA), respectively. After a rest period of three weeks following the third injection, each mouse from the immunized FAM group with the highest antibody titers selected as the donors of spleen cells for hybridoma production received a final intraperitoneal injection with the same number of conjugates in PBS. Three days later, the mice were sacrificed, and splenocytes were obtained.

5. ELISA development and data analysis

An nc-ELISA was used to determine the titers of the mouse sera and for the initial screening of hybridomas. An ic-ELISA was used to determine the sensitivity and specificity of the mouse sera and monoclonal antibodies.

nc-ELISA: Wells of microplate were coated with 100 μL of antigens in coating buffer by overnight incubation at 4 °C. Nonspecific binding was decreased by blocking the wells with a blocking solution at 37 °C for 2 h with 150 μL/well and then washed three times with a washing solution. An amount of 50 μL/well of antibody or appropriate cell culture supernatant dilutions and 50 μL of PBS was allowed to bind to the coated microwells and incubated at 37 °C for 1 h. The unbound antibody was removed by washing three times with a washing solution. Next, 100 μL/well of HRP labeled goat anti-mouse IgG (1: 5000 dilution) was added and incubated at 37 °C for 30 min. The final washing step was followed by adding 100 μL of the substrate/chromogen solution. The solution was incubated for 15 min at 37 °C and then stopped by adding 2 M H_2SO_4 (50 μL/well). The OD values of each well were measured at 450 nm.

ic-ELISA. The procedure of ic-ELISA was similar to nc-ELISA. The difference was in the addition of the antibody step. In the ic-ELISA procedure, 50 μL of antibody and 50 μL of drug standard were added to the microwell instead of 50 μL of PBS in the nc-ELISA procedure.

Triplicate experiments were performed for each concentration of competitors. Blank wells were treated in the same way as the test wells except that PBS replaced the competitor solutions. The absorbance values at 450 nm obtained in the presence of various competitor concentrations and without competitor (maximal signal) were referred to as B and B_0, respectively. The inhibition ratio was calculated as follows:

$$Inhibition\,ratio\; = \;(B\;/\;B_0)\;\times\;100\% \tag{2}$$

6. Monoclonal antibody production

Splenocytes from immunized mice were fused with SP2/0 myeloma cells using PEG1500. Fused cells were resuspended in HAT medium (RPMI minimal media supplemented with 2 mM glutamine, 1% penicillin-streptomycin, and 20% fetal bovine serum) and pipetted into the wells of 96-well plates containing a feeder layer of mouse macrophages. After one week, hybridoma supernatants were monitored by nc-ELISA and ic-ELISA, as described above. Cells in fusion wells with supernatants exhibiting the highest antibody titers and affinity when both heterologous and homologous coating antigens were transferred from the fusion plates to 24-well plates and were cloned twice by limiting dilution. For each cloning procedure, the cells were plated on a layer of macrophage feeder cells, and the final clones were obtained from rows that had been seeded with one cell per well. Finally, one part of the hybridoma was cryopreserved, and another part was collected to produce ascites via intraperitoneal injection.

The ascites collected from mice were extracted and purified by the saturated ammonium sulfate. The isotypes of the purified mAbs were then characterized with a mice mAb isotyping kit obtained from Bio-Rad Laboratories, Inc. (Hercules, CA, USA).

Table 1. Summary of reports about antibodies for very small molecules found in the literature and described in this study.

Compounds	Structures	Haptens	MW (Da)	IC_{50} (ng/mL)	Reference
Xylitol	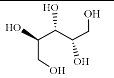	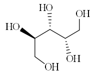	152.15	608000	[1]
3-methylindole	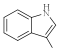	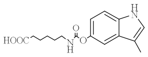	131.17	511.6~6834.3	[2]
L-hydroxyproline	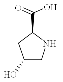	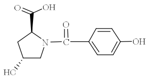	131.13	160	[3]
Melamine	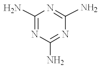	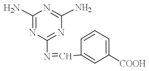	126.14	150000	[4]
Melamine	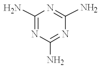	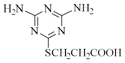	126.14	70.6	[5]
Melamine	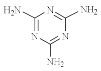	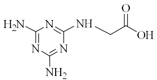	126.14	22.6	[6]
Melamine	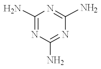	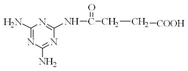	126.14	13	[7]
Melamine	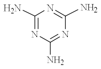	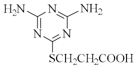	126.14	6.0	[8]
Melamine	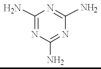	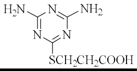	126.14	1.7	[9]

Benzoic Acid	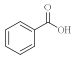	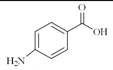	122.12	2100	[10]
Histamine	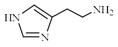	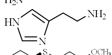	111.15	333000	[11]
Styrene	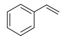	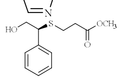	104.15	5360	[12]
Semicarbazide	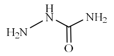	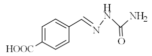	75.07	13000	[13]
Acrylamide	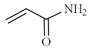	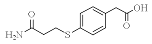	71.08	28600	[14]
Fluoroacetamide	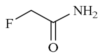	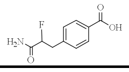	77.06	97000	This study

Table 2. The detailed information on antibodies titers and inhibition ratios of mice at the third immunizations.

**Haptens**	**FAM1**	**FAM2**	**FAM3**	**FAM4**	**FAM5**	**FAA1**	**FAA2**
Hapten	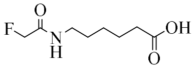	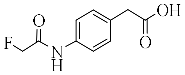	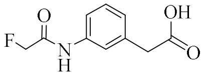	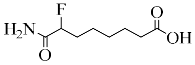	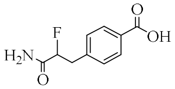	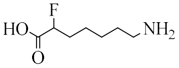	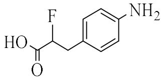
Titers ($\times 10^4$) [a]	0.69	2.63	2.38	0.63	2.75	0.50	2.19
Inhibition ratios [b]	96.48	93.57	95.22	95.75	55.68	98.31	96.33

The antibody titer is the dilution of antiserum when furnishing OD_{max} between 1.5 and 2.0 determined by nc-ELISA. Data were averaged from values of 8 mice, and each mouse was tested in triplicate.

[b] The inhibition ratio is calculated by using the following equation: $Inhibition\ ratio = (B / B_0) \times 100\%$, where B_0 and B are the responses, i.e., the OD values in the absence of FAM (B_0) and for a concentration of FAM of 500 μg/mL (B) determined by ic-ELISA. Data are averaged values of eight mice, and each mouse was tested in triplicate.

Table 3. The isotypes of mAbs.

Isotypes[b]	mAb name[a]				
	1B6	2C11	3F1	4E10	5D11
IgG_1	**2.056**[c]	**1.915**	**2.920**	**2.089**	0.190
IgG_{2a}	0.282	0.137	0.452	0.199	**1.908**
IgG_{2b}	0.206	0.083	0.335	0.156	0.068
IgG3	0.142	0.067	0.332	0.156	0.059
IgA	0.059	0.145	0.063	0.071	0.167
IgM	0.060	0.142	0.063	0.072	0.308
Kappa (κ)	**0.927**	0.249	**0.753**	**1.394**	0.562
Lambda (λ)	0.106	**1.255**	0.272	0.252	**2.817**

Note: [a]All of the mAbs were 1 mg mL^{-1} and diluted 1 × 10^4 fold.

[b] For wells with the highest response, the isotype and light chain composition are indicated.

[c] The numbers are the OD values, and the bold font highlights the antibody isotype or subtype.

References

1. Sreenath, K.; Venkatesh, Y.P. Reductively aminated D-Xylose-Albumin conjugate as the immunogen for generation of IgG and IgE antibodies specific to D-Xylitol, a haptenic allergen. *Bioconjugate Chem.* **2007**, *18*, 1995–2003, doi:10.1021/bc700175g.
2. Tuomola, M.; Harpio, R.; Mikola, H.; Knuuttila, P.; Lindstrom, M.; Mukkala, V.M.; Matikainen, M.T.; Lovgren, T. Production and characterisation of monoclonal antibodies against a very small hapten, 3-methylindole. *J. Immunol. Methods* **2000**, *240*, 111–124, doi:10.1016/s0022-1759(00)00179-4.
3. Wang, L.L.; Liu, J.X.; Liu, J.; Wang, J.P. Synthesis of hapten and production of a monoclonal antibody against a derivative of L-hydroxyproline, a special amino acid in hydrolyzed animal protein. *J. Environ. Sci. Health Part B-Pestic. Contam. Agric. Wastes* **2013**, *48*, 9–15, doi:10.1080/03601234.2012.716682.
4. Li, W.; Meng, M.; Lu, X.; Liu, W.; Yin, W.; Liu, J.; Xi, R. Preparation of anti-melamine antibody and development of an indirect chemiluminescent competitive ELISA for melamine detection in milk. *Food Agr. Immunol.* **2014**, *25*, 498–509, doi:10.1080/09540105.2013.847063.
5. Lei, H.T.; Shen, Y.D.; Song, L.J.; Yang, J.Y.; Chevallier, O.P.; Haughey, S.A.; Wang, H.; Sun, Y.M.; Elliott, C.T. Hapten synthesis and antibody production for the development of a melamine immunoassay. *Anal. Chim. Acta* **2010**, *665*, 84–90, doi:10.1016/j.aca.2010.03.007.
6. Yin, W.W.; Liu, J.T.; Zhang, T.C.; Li, W.H.; Liu, W.; Meng, M.; He, F.Y.; Wan, Y.P.; Feng, C.W.; Wang, S.L., et al. Preparation of monoclonal antibody for melamine and development of an indirect competitive ELISA for melamine detection in raw milk, milk powder, and animal feeds. *J. Agric. Food. Chem.* **2010**, *58*, 8152–8157, doi:10.1021/jf1006209.
7. Liu, J.X.; Zhong, Y.B.; Liu, J.; Zhang, H.C.; Xi, J.Z.; Wang, J.P. An enzyme linked immunosorbent assay for the determination of cyromazine and melamine residues in animal muscle tissues. *Food Control* **2010**, *21*, 1482–1487, doi:10.1016/j.foodcont.2010.04.018.
8. Sun, F.X.; Liu, L.Q.; Kuang, H.; Xu, C.L. Development of ELISA for melamine detection in milk powder. *Food Agr. Immunol.* **2013**, *24*, 79–86, doi:10.1080/09540105.2011.641170.
9. Cao, B.Y.; Yang, H.; Song, J.; Chang, H.F.; Li, S.Q.; Deng, A.P. Sensitivity and specificity enhanced enzyme-linked immunosorbent assay by rational hapten modification and heterogeneous antibody/coating antigen combinations for the detection of melamine in

milk, milk powder and feed samples. *Talanta* **2013**, *116*, 173–180, doi:10.1016/j.talanta.2013.05.009.
10. Luo, L.; Shen, Y.D.; Wang, H.; Xiao, Z.L.; Sun, Y.M.; Lei, H.T.; Yang, J.Y.; Xu, Z.L. Production of polyclonal antibody and development of a competitive enzyme-linked immunosorbent assay for benzoic acid in foods. *Food Anal. Methods* 2014, *8*, 1101–1111, doi:10.1007/s12161-014-9975-7.
11. Schneider, E.; Usleber, E.; Maertlbauer, E. Production and characterization of antibodies against histamine. In *ACS Symposium Series; Immunoassays for residue analysis: Food safety*, Beier, R.C., Stanker, L.H., Eds. American Chemical Society, Marketing Division, Room 205, 1155 16th St. N.W., Washington, DC 20036, USA: 1996; Vol. 621, pp. 413–420.
12. Shen, S.J.; Zhang, F.; Zeng, S.; Tian, Y.; Chai, X.J.; Gee, S.; Hammock, B.D.; Zheng, J. Development of enantioselective polyclonal antibodies to detect styrene oxide protein adducts. *Anal. Chem.* **2009**, *81*, 2668–2677, doi:10.1021/ac8023262.
13. Gao, A.; Chen, Q.; Cheng, Y.; Lei, J.; Zeng, L. Preparation of monoclonal antibodies against a derivative of semicarbazide as a metabolic target of nitrofurazone. *Anal. Chim. Acta* **2007**, *592*, 58–63, doi:10.1016/j.aca.2007.04.008.
14. Wu, J.; Shen, Y.D.; Lei, H.T.; Sun, Y.M.; Yang, J.Y.; Xiao, Z.L.; Wang, H.; Xu, Z.L. Hapten synthesis and development of a competitive indirect enzyme-linked immunosorbent assay for acrylamide in food samples. *J. Agric. Food. Chem.* **2014**, *62*, 7078–7084, doi:10.1021/jf5015395.
